# Interferon-stimulated genes—essential antiviral effectors implicated in resistance to Theiler’s virus-induced demyelinating disease

**DOI:** 10.1186/s12974-015-0462-x

**Published:** 2015-12-24

**Authors:** Lin Li, Reiner Ulrich, Wolfgang Baumgärtner, Ingo Gerhauser

**Affiliations:** Department of Pathology, University of Veterinary Medicine Hannover, Bünteweg 17, 30559 Hannover, Germany; Center of Systems Neuroscience Hannover, Hannover, Germany

**Keywords:** Innate immune response, Interferon-stimulated gene of 15 kDa, 2′5′-oligoadenylate synthetase, Protein kinase R, Theiler’s murine encephalomyelitis virus, Type I interferon

## Abstract

**Background:**

Experimental infection of mice with Theiler’s murine encephalomyelitis virus (TMEV) is used as an animal model of human multiple sclerosis. TMEV persists in susceptible mouse strains and causes a biphasic disease consisting of acute polioencephalomyelitis and chronic demyelinating leukomyelitis. In contrast, resistant mice eliminate the virus within 2 to 4 weeks, which seems to be based on a strong antiviral innate immune response including the activation of the type I interferon (IFN) pathway. Several interferon-stimulated genes (ISGs) such as IFN-stimulated protein of 15 kDa (ISG15), protein kinase R (PKR), and 2′5′-oligoadenylate synthetase (OAS) function as antiviral effectors and might contribute to virus elimination. Nevertheless, detailed investigations of the type I IFN pathway during TMEV-induced demyelinating disease (TMEV-IDD) are lacking.

**Methods:**

The present study evaluated microarray data of the spinal cord obtained from susceptible SJL/J mice after TMEV infection focusing on IFN-related genes. Moreover, ISG gene and protein expression was determined in mock- and TMEV-infected SJL/J mice and compared to its expression in resistant C57BL/6 mice using real- time PCR, immunohistochemistry, and immunofluorescence.

**Results:**

Interestingly, despite of increased ISG gene expression during TMEV-IDD, ISG protein expression was impaired in SJL/J mice and mainly restricted to demyelinated lesions. In contrast, high ISG protein levels were found in spinal cord gray and white matter of C57BL/6 compared to SJL/J mice in the acute and chronic phase of TMEV-IDD. In both mouse strains, ISG15 was mainly found in astrocytes and endothelial cells, whereas PKR was predominantly expressed by microglia/macrophages, oligodendrocytes, and neurons. Only few cells were immunopositive for OAS proteins.

**Conclusions:**

High levels of antiviral ISG15 and PKR proteins in the spinal cord of C57BL/6 mice might block virus replication and play an important role in the resistance to TMEV-IDD.

**Electronic supplementary material:**

The online version of this article (doi:10.1186/s12974-015-0462-x) contains supplementary material, which is available to authorized users.

## Background

Theiler’s murine encephalomyelitis virus (TMEV) is a single-stranded ribonucleic acid (RNA) (ssRNA) *Cardiovirus* of the *Picornaviridae* family, whose large open reading frame (ORF) encodes a 2300-amino acid polyprotein that is cleaved into 12 mature proteins [1]. TO strains (BeAn, DA) of TMEV induce a biphasic disease in susceptible SJL/J mice characterized by acute mild polioencephalomyelitis and chronic demyelinating leukomyelitis with virus persistence. In contrast, resistant C57BL/6 mice eliminate the virus from the central nervous system (CNS) within 2 to 4 weeks [2]. After intracerebral inoculation, TMEV primarily infects hippocampal and cortical neurons [1]. In addition, the virus spreads to neurons within the ventral horns of the spinal cord most likely along axons and via the cerebrospinal fluid [3, 4]. Approximately 2 weeks after inoculation at the peak of the specific immune response, the virus disappears from neurons but persists in white matter glial cells and macrophages [5]. Demyelination is initially induced by virus-mediated axonal damage recruiting inflammatory cells to sites of Wallerian-like degeneration [6]. The subsequent release and presentation of myelin breakdown products causes a CD4^+^ T cell response directed against different myelin proteins such as proteolipid protein, epitope PLP_139-151_, by molecular mimicry and epitope spreading [7]. The susceptibility of mouse strains to TMEV-induced demyelination disease (TMEV-IDD) is linked to the major histocompatibility complex (MHC) class I H-2D gene [8]. Consequently, the level of TMEV-specific CD8^+^ cytotoxic T cells is higher in resistant compared to susceptible mice in acute lesions [9]. In addition, MHC class II-restricted CD4^+^ T cells play an important role in the induction of a delayed-type hypersensitivity (DTH) response initially directed against specific TMEV capsid proteins [10].

Due to similar histological and pathogenetic features, TMEV-infected susceptible mice are commonly used to model the most important demyelinating disease in humans multiple sclerosis (MS) [3, 11]. The therapy of MS strongly relies on different immunomodulatory and immunosuppressive drugs including beta interferon (IFN-β), which blocks T cell activation, increases interleukin (IL)-10 expression, and maintains blood-brain barrier integrity [12, 13]. Similar IFN-β-dependent mechanisms seem to reduce the demyelination in chronic TMEV-IDD [7]. Furthermore, antiviral actions of this type I interferon (IFN) have to be blocked by the TMEV leader (L) protein, a 76-amino acid protein cleaved from the N-terminal end of viral polyprotein, to establish virus persistence [14]. The L protein impedes IFN-β gene transcription by interfering with IFN regulatory factor 3 (IRF3) dimerization and nucleocytoplasmic trafficking [2, 15, 16]. Interestingly, IRF3 contributes not only to early control of TMEV replication but also to TMEV-induced hippocampal injury [17]. In addition, IRF3 polymorphisms strongly affect the murine innate immune response and ability of TMEV to persist in the CNS of different mouse strains [18]. Consequently, TMEV-induced demyelination might result from an insufficient infection control by the innate immune response particularly the interferon system in susceptible mice.

Type I IFN signal transduction is initiated by their cell surface receptors, IFN alpha and beta receptors (IFNAR)1 and 2, which are linked to janus kinase 1 (JAK1) and tyrosine kinase 2 (TYK2). These two tyrosine kinases activate the IFN-stimulated gene factor 3 (ISGF3) consisting of the signal transducer and activator of transcription (STAT) 1 and 2 and IRF9, which enter the nucleus and bind to IFN-stimulated response elements (ISREs) of more than 300 IFN-stimulated genes (ISGs) [19, 20]. The death of IFNAR knockout mice after TMEV infection supports the prominent role of ISGs in virus control [21]. IFN-stimulated protein of 15 kDa (ISG15), protein kinase R (PKR), 2′5′-oligoadenylate synthetase (OAS), and myxovirus resistance (Mx) represent classical IFN-dependent antiviral effectors detected by gene targeting studies [19]. In contrast to other ISGs, the effector functions of these proteins have been characterized in detail. In addition, studies using specific knockout mice have confirmed their antiviral activity against numerous virus families including *Picornaviridae* [22, 23]. Consequently, mouse strain-specific differences in their expression pattern might critically influence their ability to eliminate TMEV from the CNS. ISG15 encodes an ubiquitin-like protein, which conjugates to numerous protein substrates to modulate pleiotropic cellular responses [21, 24]. PKR is activated by viral double-stranded RNA (dsRNA) resulting in phosphorylation of the eukaryotic initiation factor 2α (eIF2α) and inhibition of global protein synthesis including viral protein synthesis [19, 25]. OAS proteins including OAS1, OAS2, OAS3, and OASL (OAS-like) are expressed at low levels in unstimulated cells as inactive monomers, which can be activated by viral dsRNA to form tetramers [26]. These tetramers activate the latent ribonuclease L (RNase L) resulting in the degradation of viral and host cell RNAs [19, 26]. Mx proteins are members of the dynamin-like large guanosine triphosphatase (GTPase) family, whose expression is exclusively controlled by type I IFNs [27]. Mx proteins bind viral nucleocapsid-like structures or other viral components to prevent the generation of new virus particles [27]. However, Mx proteins are not functional in most laboratory mouse strains [28, 29] but might be biomarkers to predict the therapeutic efficacy of IFN-β treatment in MS [30].

The role of these classical ISGs in the neuropathogenesis of TMEV-IDD has already been investigated in previous studies. PKR is important for the recognition of TMEV RNA in the cytoplasm of astrocytes [31] and the ensuing expression of several pro-inflammatory cytokines such as IL-1, IL-6, IL-12p40, and TNF-α as well as chemokines including different monocyte chemoattractant proteins [32, 33]. In addition, PKR is required for production of type I IFNs in response to TMEV in part by regulating IFN messenger RNA (mRNA) integrity [34]. The OAS/RNase L pathway has been described to be inhibited by the direct interaction of the ankyrin domain of RNase L with TMEV L* protein, a 156-amino acid long protein encoded by an alternative TMEV ORF [35]. Surprisingly, primary mouse neurons that are exposed to type I IFNs remain highly susceptible to TMEV infection, which results from their heterogeneous response to IFN treatment revealed by a lack of Mx1 expression. In addition, they have a low level of basal ISG expression compared to fibroblasts [36]. These data underline the cell type-specific differences in their IFN response. However, all these studies have been performed in cell culture and have not investigated the expression of these antiviral effectors at the protein level. Moreover, ISG cell type-specific expression patterns in the spinal cord including white matter lesions of TMEV-infected mice remain unknown so far. Consequently, the aim of this study was to compare the expression of these four important ISGs in TMEV-IDD between SJL/J and C57BL/6 mice in order to substantiate the hypothesis that intrinsic differences in the interferon system between these mouse strains are related to their susceptibility to TMEV-induced demyelination.

## Methods

### Animals and Virus infection

Twenty-four 5-week-old female SJL/J HanHsd mice and 11 C57BL/6 mice (Harlan Winkelmann, Borchen, Germany) were kept in a microisolator cage system (Tecniplast, Hohenpeißenberg, Germany) with standard bedding material (ssniff Spezialdiäten GmbH, Soest, Germany) under a 12:12 h light/dark cycle. Food (R/M-H V1534; ssniff Spezialdiäten GmbH) and water were available ad libitum. They were inoculated into the right cerebral hemisphere with 1.63 × 10^6^ plaque-forming units (PFU) per mouse of the BeAn strain of TMEV in 20 μl Dulbecco’s modified Eagle medium (DMEM; PAA Laboratories, Cölbe, Germany) with 2 % fetal calf serum and 50 μg/kg gentamycin under general anesthesia using medetomidine (0.5 mg/kg, Domitor; Pfizer, Karlsruhe, Germany) and ketamine (100 mg/kg, Ketamin 10 %; WDT eG, Garbsen, Germany). Twenty-four 5-week-old female SJL/J HanHsd and 12 C57BL/6 mice were mock-infected using 20 μl DMEM only. TMEV- and mock-infected mice were sacrificed in groups of five to six animals at 14, 42, 98, and 196 days post infection (dpi), respectively. Euthanasia was performed with a fivefold overdose of the described anesthetics. Segments of the spinal cord of mice were removed immediately after death and either formalin-fixed and paraffin-embedded or snap-frozen in OCT embedding compound (Sakura Finetek Europe, Zoeterwoude, Netherlands) using liquid nitrogen [37]. Microarray analysis was performed on 23 TMEV- (5 mice at 98 dpi; 6 mice at 14, 42, and 196 dpi) and 24 mock-infected SJL/J mice (6 mice at all time points). Spinal cord tissue obtained from these 47 SJL/J mice was also studied using immunohistochemistry at all four time points. ISG immunoreactivity and transcript numbers of selected genes were compared between SJL/J and C57BL/6 mice at 14 dpi (6 mock- and 6 TMEV-infected mice, respectively) and 98 dpi (6 mock- and 5 TMEV-infected mice, respectively) using immunohistochemistry and reverse transcription quantitative polymerase chain reaction (RT-qPCR), respectively. Moreover, immunofluorescence was performed on one additional TMEV-infected SJL/J mouse due to the lack of adequate tissue from the other SJL/J mice. Animal experiments were authorized by local authorities (Niedersächsisches Landesamt für Verbraucherschutz- und Lebensmittelsicherheit, Oldenburg, Germany, permission numbers: 509c-42502-02/589, 509.6-42502-04/860, 33-42502-05/963, 33-42502-04-07/1292).

### Microarray analysis

For microarray analysis, RNA was isolated from snap-frozen spinal cord specimens using the RNeasy Mini Kit (Qiagen, Hilden, Germany), amplified and labeled employing the MessageAmp II Biotin Enhanced Kit (Ambion, Austin, TX, USA), and hybridized to GeneChip mouse genome 430 2.0 arrays (Affymetrix, Santa Clara, CA, USA) as previously described [38, 39]. Background adjustment and quantile normalization was performed using RMAexpress [40]. MIAME compliant data sets are deposited in the ArrayExpress database (E-MEXP-1717; http://www.ebi.ac.uk/arrayexpress). Bottom-up analysis of manually selected candidate genes was done using classical statistics as previously described [41–43]. Accordingly, 111 candidate genes comprising 10 pattern recognition receptors (PRRs), 10 IFN regulatory factors, 9 type I/II IFNs, 4 type I/II IFN receptors, 8 signal transducers, 60 IFN-dependent antiviral effectors, and 10 MHC class I/II genes were manually extracted from peer-reviewed published literature (Additional file 1: Table S1) [19, 22, 23, 44, 45]. The fold change was calculated as the ratio of the inverse-transformed arithmetic means of the log2-transformed expression values of TMEV-infected vs. mock-infected mice. Downregulations are shown as negative reciprocal values [39, 46]. The normalized expression values of these genes were evaluated for significant differences between TMEV- and mock-infected mice employing independent pairwise Mann-Whitney *U* tests (Prism 6, GraphPad Software, La Jolla, CA, USA). Statistical significance was designated as *P* ≤ 0.05.

### Polymerase chain reaction

RT-qPCR was performed for IFN-α (using consensus primers annealing with all IFN-α subtypes), IFN-β, IRF-7, ISG15, PKR, and three housekeeping genes (GAPDH, β-Actin, HPRT) using standard protocols, the Mx3005P qPCR System (Agilent Technologies Deutschland GmbH, Böblingen, Germany), and Brilliant III Ultra-Fast SYBR® QPCR Master Mixes [47]. The following primers were used: IFN-α *forward* ATGGCTAGRCTCTGTGCTTTCCT; IFN-α *reverse* AGGGCTCTCCAGAYTTCTGCTCTG; IFN-β *forward* TGAATGGAAAGATCAACCTCACCTA; IFN-β *reverse* CTCTTCTGCATCTTCTCCGTCA; IRF7 *forward* CGAGTGCTGTTTGGAGACTG; IRF7 *reverse* GGCCTTGAAGATCTGTGCAT; ISG15 *forward* AACTGCAGCGAGCCTCTGA; ISG15 *reverse* CACCTTCTTCTTAAGCGTGTCTACAG; PKR *forward* GTACAAGCGCTGGCAGAACTCAAT; and PKR *reverse* AAGAGGCACCGGGTTTTGTAT. Tenfold serial dilution standards ranging from 10^8^ to 10^2^ copies/μL were used to quantify the results. A normalization factor achieved from the housekeeping genes was calculated to correct for experimental variations [48]. Specificity of each reaction was controlled by melting curve analysis. Similar to microarray analysis, fold changes were calculated to compare the respective results.

### Histology and immunohistochemistry

Two- to three-micrometer paraffin spinal cord sections of SJL/J mice at 14, 42, 98, and 196 dpi and C57BL/6 mice at 14 and 98 dpi were routinely stained with hematoxylin and eosin (HE) or used for immunohistochemistry as described [47]. Briefly, dewaxed and rehydrated sections were incubated in ethanol with 0.05 % hydrogen peroxide for 30 min to block endogenous peroxidase. Antigen retrieval was performed for the detection of PKR (boiled in 10 mM citrate buffer, 15 min, pH 6.0) and ISG15 and OAS1 (trypsin solution with CaCl_2_ × 2 H_2_O, 20 min, pH 7.8). Subsequently, sections were blocked with phosphate-buffered saline (PBS) containing 20 % horse serum (Mx1/2/3) or 20 % goat serum (PKR, ISG15, OAS1) for 30 min at room temperature. Sections were incubated with an affinity-purified goat polyclonal anti-Mx1/2/3 antibody (sc-34130, Santa Cruz Biotechnology, Santa Cruz, CA, USA; 1:100), a rabbit monoclonal anti-PKR antibody (ab-32036, Abcam, Cambridge, MA, USA; 1:400), or rabbit polyclonal antibodies directed against ISG15 (sc-50366, Santa Cruz Biotechnology; 1:200) or OAS1 (sc-98424, Santa Cruz Biotechnology,; 1:400). Negative control sections were incubated with goat serum (G9023, Sigma-Aldrich, Taufkirchen, Germany; 1:5650) and rabbit serum (R4505, Sigma-Aldrich) diluted at the same gamma globulin concentration as the primary antibodies. Biotinylated horse-anti-goat antiserum (BA-9500, Vector Laboratories, Burlingame, CA, USA; 1:200) and goat-anti-rabbit antiserum (BA-1000, Vector Laboratories; 1:200) were used as secondary antibodies, and immunolabeling was visualized by the avidin-biotin-peroxidase complex (ABC) method (PK-6100, Vector Laboratories) with 3,3-diaminobenzidine (DAB) as substrate. Finally, sections were slightly counterstained with Mayer’s hematoxylin.

Photos of ISG15, PKR, and OAS1 immunohistochemically stained sections were generated by a digital microscope (HS All-in-one Fluorescence Microscope BZ-9000 Generation II; HS All-in-one Fluorescence Microscope BZ-II Analyzer, BIOREVO, KEYENCE Deutschland GmbH, Neu-Isenburg, Germany) and evaluated using analysis software (analySIS 3.1 software package; Soft Imaging system, Münster, Germany). Light microscopy was used to count Mx-positive cells. In addition, ISG positive and negative cells present in the Virchow-Robin space of meningeal and intraparenchymal blood vessels were counted to calculate the percentage of ISG15-, PKR-, OAS1-, and Mx-positive perivascular cells. Immunohistochemical results are expressed as median with minimum and maximum.

### Immunofluorescence double-labeling

Immunofluorescence was used to identify cells expressing ISG15 and PKR as described [49, 50]. Briefly, after citrate buffer heat-mediated antigen retrieval and incubation with PBS containing 20 % horse serum or 20 % goat serum to block non-specific binding, paraffin sections were co-incubated with antibodies directed against ISG15 (sc-50366; 1:100) or PKR (ab-32036; 1:200) and antibodies directed against GFAP (ab-3554, Abcam; 1:200), Olig2 (ab-85900, Abcam; 1:500), or CD107b (MCA2293, AbD Serotec, Puchheim, Germany; 1:200) overnight at 4 °C. Negative controls included sections incubated with rabbit serum (R4505, Sigma-Aldrich; 1:3000/1:6000), goat serum (I9140, Sigma-Aldrich; 1:4500), sheep serum (1:5000), and rat serum (R9759, Sigma-Aldrich; 1:16000), respectively. After washing, sections were incubated Cy2- and Cy3-conjugated secondary antibodies (goat anti-rabbit, A-11034, Invitrogen; 1:200; goat anti-rat, 112-165-003; donkey anti-rabbit, 711-545-152; donkey anti-sheep, 713-765-147; donkey anti-goat, 705-765-147, Jackson ImmunoResearch, Suffolk, UK; 1:200) in the dark for 1 h at room temperature. Nuclei were counterstained with 0.01 % bisbenzimide (H33258, Sigma-Aldrich), and sections were mounted in Dako Fluorescence Mounting Medium (S3023, DakoCytomation GmbH, Hamburg, Germany).

### Statistical analysis

RT-qPCR data and immunohistochemical data of SJL/J and C57BL/6 mice were analyzed using Kruskal-Wallis and non-parametric Mann-Whitney *U* tests (IBM SPSS, version 19.0, IBM Corporation, Armonk, NY, USA). Ratios of the geometric means and 95 % confidence intervals were calculated with independent *T* tests to compare ISG protein expression between C57BL/6 and SJL/J mice (IBM SPSS, version 19.0). Spearman’s rank correlation coefficients were calculated to analyze the correlation between ISG protein and gene expression as well as TMEV RNA copy numbers, inflammation, and demyelination in the spinal cord of SJL/J mice (IBM SPSS, version 19.0). Data concerning TMEV, inflammation (determined on hematoxylin and eosin and immunohistochemically stained sections), and demyelination (determined on luxol fast blue stained sections) have been taken from previous publications [39, 51]. Statistical significance was designated as *P* ≤ 0.05.

## Results

### Microarray

**Fig. 1:**
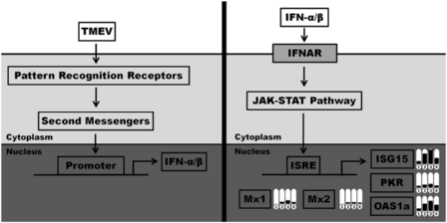
Type I IFN signaling pathway and transcriptional changes of selected ISGs in the spinal cord of TMEV- compared to mock-infected SJL/J mice. *Left side*: Pattern recognition receptors (PKR, RIG-I-like helicases, Toll-like receptors) recognize viral proteins and RNA, which induce the activation of second messengers (IFN regulatory factors, NF-κB). These signal transducers translocate into the nucleus and induce the transcription of IFNα/β. *Right side*: These type I IFNs bind to the type I IFN receptor (IFNAR) to initiate signal transduction via the JAK-STAT pathway. This pathway activates specific transcription factors (ISGF3), which translocate into the nucleus, bind to IFN-stimulated response element (ISRE) of the DNA, and induce the transcription of abundant ISGs including ISG15, PKR, OAS, and Mx. Thermometer-like icons display significant fold changes (*P* ≤ 0.05) at four time points (1 = 14 dpi, 2 = 42 dpi, 3 = 98 dpi, 4 = 196 dpi). Legend: *IFN* interferon, *ISG* IFN-stimulated gene, *ISG15* ISG of 15 kDa, *ISGF3* ISG factor 3, *JAK* janus kinase, *Mx* myxovirus-resistance gene, *NF-κB* nuclear factor of kappa light polypeptide gene enhancer in B cells, *OAS* 2′, 5′-oligoadenylate-synthetase, *PKR* protein kinase R, *RIG-I* retinoic acid-inducible gene, *STAT* signal transducer and activator of transcription, *TMEV* Theiler’s murine encephalomyelitis virus

Recently, Ulrich et al. (2010) performed a microarray analysis of mock- and TMEV-infected SJL/J mice at 14, 42, 98, and 196 dpi and detected transcriptional changes of 1001 genes in the spinal cord. In the present study, the log2-transformed normalized data were independently re-evaluated focusing on critical genes of the type I IFN pathway employing conventional statistical methods as described [41, 43, 52]. Most genes were significantly up-regulated by TMEV infection and peaked at 98 dpi (Fig. 1; Table 1; Additional file 1: Table S1). Only a mild increase in Toll-like receptor (Tlr)3 gene expression (fold change <2) was found, whereas other receptors of viral RNA including Pkr, melanoma differentiation-associated gene (Mda5), Tlr7, and Tlr13 were moderately up-regulated (fold change up to 5.77). Nfkb1, lrf3, and Irf5 gene expression was barely influenced by TMEV infection. In contrast, Irf1 and Irf7 were strongly up-regulated (fold change up to 4.40 and 11.42, respectively). Interestingly, no changes were found in type I IFN gene expression, and Ifnar genes were only mildly increased in the late phase of TMEV-IDD. However, gene expression of several factors critically involved in IFN signal transduction, especially of the ISGF3 components IRF9, STAT1, and STAT2, was moderately to strongly up-regulated (fold change up to 15.39). In addition, gene expression of various IFN-dependent antiviral effectors was strongly increased in TMEV-IDD (fold change up to 31.22). Similarly, several MHC class I and II genes were strongly induced by TMEV infection (fold change up to 37.55).

**Table 1:** Transcriptional changes of the type I IFN signaling pathway in TMEV- compared to mock-infected SJL/J mice

Gene symbol	14 dpi	42 dpi	98 dpi	196 dpi
Pattern recognition receptors				
Eif2ak2 (Pkr)	*1.69*	*3.04*	*3.73*	*2.18*
Ifih1 (Mda5)	*2.20*	*4.86*	*5.77*	*3.51*
Tlr2	*1.77*	*2.66*	*3.31*	*2.94*
Tlr3	1.18	*1.37*	*1.89*	*1.38*
Tlr7	1.18	*1.87*	*2.47*	*2.43*
Tlr13	*1.33*	*2.50*	*3.46*	*3.27*
Interferon regulatory factors				
Irf1	*1.51*	*3.47*	*4.40*	*3.11*
Irf3	1.07	1.06	1.09	*1.18*
Irf5	1.07	*1.40*	*1.81*	*1.57*
Irf7	*2.66*	*10.12*	*11.42*	*4.46*
Nfkb1	*1.14*	*1.14*	*1.20*	*1.10*
Type I interferons				
Ifna1	1.04	−1.10	−1.06	−1.08
Ifnb1	1.02	1.07	−1.08	−1.11
Type I interferon receptors				
Ifnar1	1.01	−1.02	*1.14*	*1.15*
Ifnar2	1.10	*1.51*	*1.72*	*1.50*
Signal transducers				
Irf9	*2.53*	*4.92*	*4.72*	*3.21*
Jak1	1.01	1.02	−1.04	*1.06*
Socs1	1.01	*1.31*	1.48	1.14
Stat1	*3.26*	*10.58*	*15.39*	*9.57*
Stat2	*1.39*	*2.49*	*3.30*	*2.03*
Tyk2	1.15	*1.12*	*1.32*	*1.19*
Classical interferon stimulated genes				
Isg15	*3.23*	*11.58*	*15.71*	*6.59*
Mx1	1.34	*2.11*	*3.85*	*1.88*
Mx2	1.24	*1.61*	*1.76*	*1.31*
Oas1a	*2.71*	*7.92*	*11.07*	*6.73*
Oas2	*1.32*	*2.26*	*2.59*	*2.08*
Oas3	1.01	*1.59*	*1.75*	1.23
Oasl1	*1.61*	*2.81*	*3.65*	*1.99*
Oasl2	*6.70*	*25.78*	*29.16*	*12.93*
Rnasel	−1.16	1.37	*1.54*	*1.29*
Major histocompatibility (MHC) genes class I/II				
H2-D1 (MHC-Ia)	*5.58*	*8.03*	*9.78*	*9.42*
H2-K1 (MHC-Ia)	*5.42*	*7.45*	*9.70*	*8.56*
H2-M3 (MHC-Ib)	*1.62*	*2.80*	*3.48*	*3.25*
H2-Q5 (MHC-Ib)	*2.46*	*9.75*	*13.88*	*10.7*
H2-Aa (MHC-IIa)	*5.49*	*29.30*	*37.55*	*29.10*
H2-Eb1 (MHC-IIa)	*5.11*	*20.11*	*28.18*	*21.00*
H2-DMa (MHC-IIb)	*1.31*	*2.93*	*4.23*	*3.22*
H2-Oa (MHC-IIb)	*1.25*	*2.78*	*4.39*	*2.79*

Shown are fold changes at 14, 42, 98, and 196 dpi. Italic type indicates statistically significant up-regulation (*P* ≤ 0.05)

Legend: *Eif2ak2* eukaryotic translation initiation factor 2-alpha kinase 4, *H2-Aa* H2 antigen A alpha, *H2-D1* H2-D region locus 1, *H2-DMa* H2 antigen locus DMa, *H2-Eb1* H2 antigen E beta 1, *H2-K1* H2-K region locus 1, *H2-M3* H2-M region locus 3, *H2-Oa* H2-O region alpha locus, *H2-Q5* H2-Q region locus 5, *Ifih* interferon induced with helicase C domain, *Ifit* interferon-induced protein with tetratricopeptide repeats, *Ifna* interferon alpha, *Ifnb* interferon beta, *Ifnar* interferon (alpha and beta) receptor, *Irf* interferon regulatory factor, *Isg* interferon-stimulated protein, *Jak* Janus kinase, *Mx* Myxovirus (influenza virus) resistance, *Nfkb* nuclear factor of kappa light polypeptide gene enhancer in B cells, *Oas* 2′-5′ oligoadenylate synthetase, *Rnasel* Ribonuclease L (2′, 5′-oligoisoadenylate synthetase-dependent), *Socs* suppressor of cytokine signaling, *Stat* signal transducer and activator of transcription, *Tlr* Toll-like receptor, *Tyk* tyrosine kinase

### RT-qPCR

RT-qPCR of selected genes was performed to validate microarray results of SJL/J mice and to compare gene expression of SJL/J and C57BL/6 mice (Additional file 2: Figure S1). In general, there was a good correlation between RT-qPCR and microarray data in SJL/J mice revealing highly similar fold changes of IRF7, ISG15, and PKR at both 14 and 98 dpi. Nevertheless, minor changes in type I IFN mRNA transcription were detected in SJL/J mice using RT-qPCR, which were not found in the microarray analysis.

IFN-α and IFN-β transcript numbers were not significantly changed at 14 dpi in SJL/J mice (fold changes: IFN-α: 1.33, IFN-β: −1.07). At 98 dpi mRNA levels of both type I IFNs were downregulated (fold changes: IFN-α: −2.12, IFN-β: −2.78), but only IFN-β differed significantly (IFN-α: *P* = 0.052, IFN-β: *P* = 0.030). IRF7, ISG15, and PKR were significantly up-regulated at 14 dpi (fold changes: IRF7: 4.96 (*P* = 0.009), ISG15: 4.13 (*P* = 0.009), PKR: 1.64 (*P* = 0.026)) and 98 dpi (fold changes: IRF7: 11.53 (*P* = 0.017), ISG15: 21.12 (*P* = 0.004), PKR: 3.19 (*P* = 0.004)) in SJL/J mice.

C57BL/6 mice showed a significant increase in mRNA levels of IFN-β at 14 dpi (fold change 3.73, *P* = 0.041) and of IFN-α at 98 dpi (fold change 2.49, *P* = 0.017). TMEV infection did not significantly influence the transcript numbers of IFN-β at 98 dpi (fold change 1.42, *P* = 0.247) and of IFN-α at 14 dpi (fold change −1.01, *P* = 1.000). Similar to IFN-β, ISG15 was up-regulated at 14 dpi (fold change 2.11, *P* = 0.026) but not at 98 dpi (fold change 1.59, *P* = 0.177) in C57BL/6 mice. Statistical analysis detected a tendency of increased IRF7 mRNA levels at both time points (fold changes: 14 dpi: 2.24, *P* = 0.093; 98 dpi: 2.02, *P* = 0.052). PKR transcript numbers were not significantly influenced by TMEV infection in this mouse strain (fold changes: 14 dpi: 1.26, 98 dpi: 1.06).

Significant differences between the two mouse strains at the mRNA levels of the investigated genes were predominantly found at 98 dpi. In mock-infected mice, there were higher transcript numbers of IFN-α (*P* = 0.041) and IFN-β (*P* = 0.009) and lower mRNA levels of ISG15 (*P* = 0.009) in SJL/J compared to C57BL/6 mice at this time point. In TMEV-infected mice, there were higher transcript numbers of IRF7 (*P* = 0.008), ISG15 (*P* = 0.008), and PKR (*P* = 0.008) and lower mRNA levels of IFN-α (*P* = 0.016) in SJL/J compared to C57BL/6 mice at 98 dpi. At 14 dpi, a significant difference between the two mouse strains was only detected for IFN-β, which was higher in mock- (*P* = 0.002) and TMEV-infected (*P* = 0.002) SJL/J compared to C57BL/6 mice.

### Immunohistochemistry

#### TMEV infection induces ISG protein expression in SJL/J mice

**Fig. 2:**
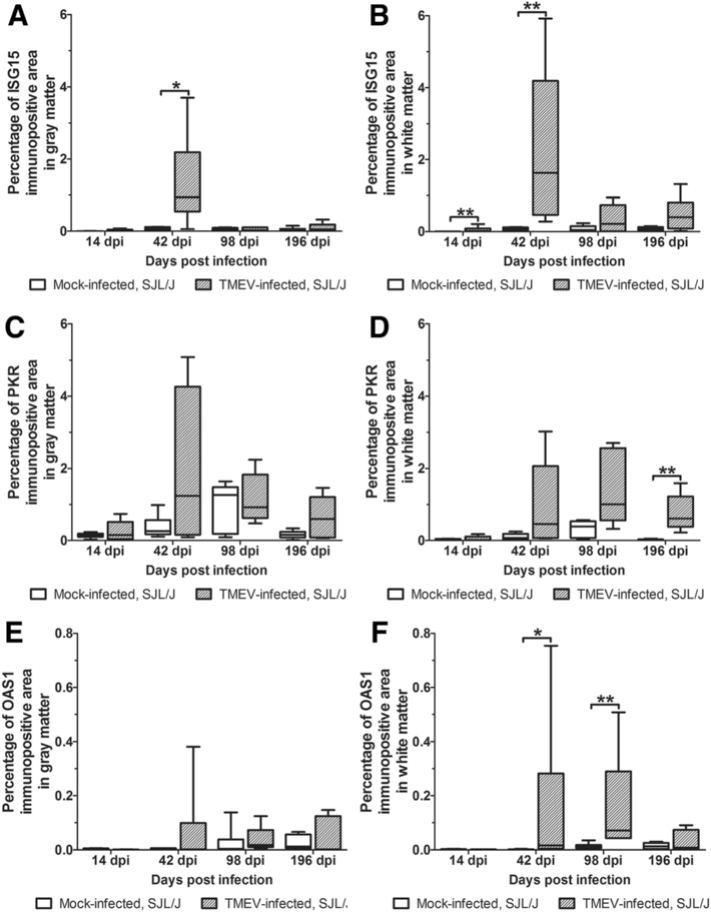
ISG15, PKR, and OAS1 protein expression in the spinal cord of mock- and TMEV-infected SJL/J at 14, 42, 98, and 196 dpi. ISG15 (**a**, **b**), PKR (**c**, **d**), and OAS1 (**e**, **f**) protein expression in spinal cord gray (**a**, **c**, **e**) and white matter (**b**, **d**, **f**). Shown is the percentage of immunopositive area using Box-and-Whisker plots and significant differences between groups based on Mann-Whitney *U* tests (*P ≤ 0.05; **P ≤ 0.01)

**Fig. 3:**
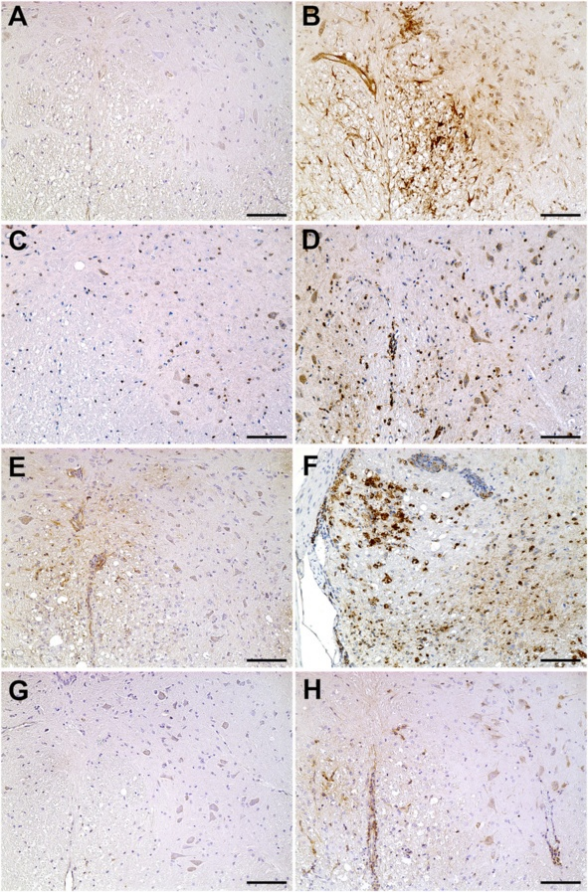
ISG15, PKR, and OAS1 protein expression in the spinal cord of mock- and TMEV-infected SJL/J mice. **a** Low expression of ISG15 proteins in mock-infected mice at 42 dpi. **b** High expression in endothelial cells and astrocytes of TMEV-infected SJL/J mice at 42 dpi. **c** Few cells express PKR proteins in mock-infected SJL/J mice at 196 dpi. **d** Many oligodendrocytes, microglia/macrophages, and neurons express PKR proteins in TMEV-infected SJL/J mice at 196 dpi. **e** Few intralesional cells express ISG15 at 98 dpi. **f** High PKR expression in intralesional gitter cells and some perivascular immune cells in TMEV-infected SJL/J mice at 98 dpi. **g** Very low expression of OAS1 proteins of mock-infected SJL/J mice at 98 dpi. **h** Few neurons, microglia, and perivascular immune cells express OAS1 proteins in TMEV-infected SJL/J mice at 98 dpi. Immunohistochemistry visualized by the avidin-biotin-peroxidase complex method with 3,3-diaminobenzidine as substrate and counterstaining with Mayer’s hematoxylin. Bar 100 μm

In general, immunohistochemical analysis detected an increase in ISG15, PKR, and OAS1 protein expression in white matter lesions (Figs. 2 and 3). ISG15-positive cells particularly ependymal cells, endothelial cells, astrocytes, and microglia/ microphages were found in the spinal cord of mock- and TMEV-infected SJL/J mice at all of time points. A mild ISG15 expression was also found in some neurons (Fig. 3a, b, e). ISG15 protein levels were higher in the white matter of TMEV- compared to mock-infected mice at 14 dpi (*P* = 0.004) and 42 dpi (*P* = 0.002), whereas protein expression was only changed at 42 dpi in the gray matter *(P* = 0.015; Fig. 2a, b).

PKR proteins were present in microglia/macrophages, oligodendrocytes, and neurons at all investigated time points in mock- and TMEV-infected mice (Fig. 3c, d). In addition, positive gitter cells were found in the white matter of TMEV-infected SJL/J mice at 98 and 196 dpi (Fig. 3f). Similar to ISG15, PKR protein levels were increased by TMEV infection, but a significant difference was only found in the white matter at 196 dpi due to strong intralesional expression (*P* = 0.002; Figs. 2d and 3e, f).

OAS1 was mainly expressed by neurons and microglia in mock- and TMEV-infected mice (Fig. 3g, h). In general, there was a low OAS1 protein expression at all investigated time points (Fig. 2e, f). Nevertheless, OAS1 protein levels were significantly higher in the white matter of TMEV- compared to mock-infected mice at 42 dpi (*P* = 0.026) and 98 dpi (*P* = 0.004; Fig. 2f). Corresponding to low IFNα and IFNβ gene expression, Mx proteins were only found in few glial cells at 14 and 98 dpi.

#### Higher ISG protein expression in C57BL/6 compared to SJL/J mice

The ISG protein expression in the spinal cord was compared between susceptible SJL/J and resistant C57BL/6 mice at an early time point (14 dpi) and at the peak of inflammatory demyelination and ISG gene induction (98 dpi). Similar to SJL/J mice, only few Mx-positive cells were detected in mock- and TMEV-infected C57BL/6 mice indicating comparable low type I IFN levels in both mouse strains. In contrast, ISG15 protein levels were significantly increased in TMEV- compared to mock-infected C57BL/6 mice in the gray (*P* = 0.026) and white matter (*P* = 0.009) at 14 dpi and in the gray matter (*P* = 0.017) at 98 dpi (Additional file 3: Figure S2). PKR protein levels were significantly up-regulated in TMEV- compared to mock-infected C57BL/6 mice in the gray (*P* = 0.030) and white matter (*P* = 0.004) only at 98 dpi. OAS1 protein expression was not influenced significantly by TMEV infection in C57BL/6 mice.

**Fig. 4:**
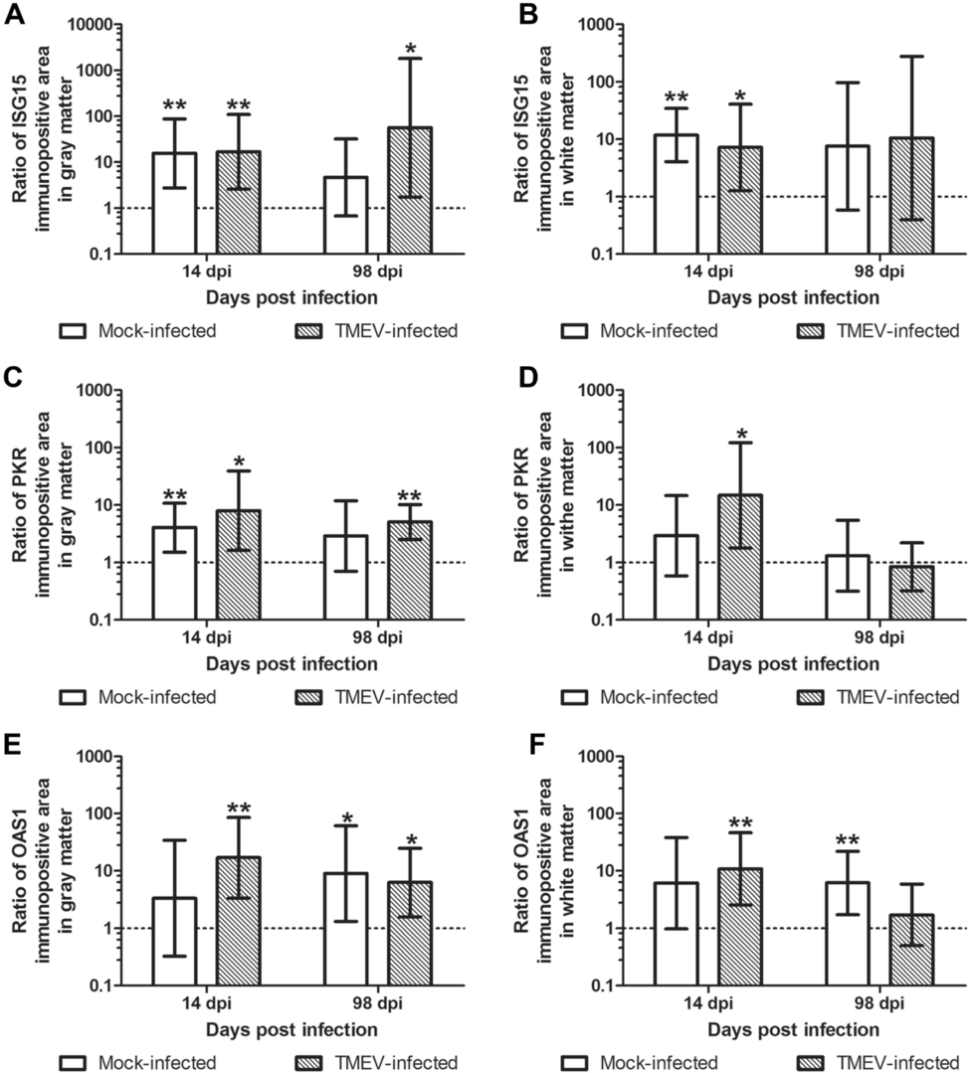
Comparison of the ISG15, PKR, and OAS1 protein expression between mock- and TMEV-infected C57BL/6 and SJL/J mice at 14 and 98 dpi. ISG15 (**a**, **b**), PKR (**c**, **d**), and OAS1 (**e**, **f**) protein expression in spinal cord gray (**a**, **c**, **e**) and white matter (**b**, **d**, **f**). Shown are the ratios of the geometric means and 95 % confidence intervals of immunopositive area between C57BL/6 and SJL/J mice using Box-and-Whisker plots and significant differences between groups based on independent *T* tests (*P ≤ 0.05; **P ≤ 0.01). Note higher protein levels in C57BL/6 compared to SJL/J mice (ratio >1)

**Fig. 5:**
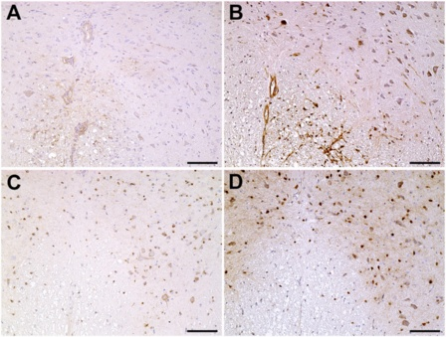
ISG15 and PKR protein expression in the spinal cord of mock- and TMEV-infected C57BL/6 mice. **a** Few cells express ISG15 proteins in TMEV-infected SJL/J mice at 98 dpi. **b** Many endothelial cells, astrocytes, and neurons express ISG15 proteins in TMEV-infected C57BL/6 mice at 98 dpi. **c** Low expression of PKR proteins in TMEV-infected SJL/J mice at 98 dpi. **d** High expression of PKR proteins in oligodendrocytes, microglia/macrophages, and neurons of TMEV-infected C57BL/6 mice at 98 dpi. Immunohistochemistry visualized by the avidin-biotin-peroxidase complex method with 3,3-diaminobenzidine as substrate and counterstaining with Mayer’s hematoxylin. Bar 100 μm

However, significant higher ISG15, PKR, and OAS1 protein expression was detected in the spinal cord gray matter of TMEV-infected C57BL/6 compared to SJL/J mice at 14 dpi (*P* = 0.007, *P* = 0.016, *P* = 0.003) and 98 dpi (*P* = 0.032, *P* = 0.001, *P* = 0.025; Figs. 4 and 5). Significant higher ISG15, PKR, and OAS1 protein levels in the spinal cord white matter of TMEV-infected C57BL/6 compared to SJL/J mice were only found at 14 dpi (*P* = 0.029, *P* = 0.017, *P* = 0.004). Furthermore, there was a significant higher ISG15 expression of mock-infected C57BL/6 compared to SJL/J mice in spinal cord gray (*P* = 0.005) and white matter (*P* < 0.001) at 14 dpi (Fig. 4). PKR and OAS1 protein levels were also significantly increased in mock-infected C57BL/6 mice in the gray matter at 14 dpi (*P* = 0.01) and in the white matter at 98 dpi (*P* = 0.01), respectively. At 14 dpi, the difference in OAS1 expression in spinal cord white matter between the two mouse strains after mock-infection did not reach statistical significance (*P* = 0.052).

A perivascular infiltration of lymphocytes, plasma cells, and macrophages was only found in TMEV-infected mice. Few perivascular cells (<10 %) expressed OAS1 or Mx proteins in both mouse strains. ISG15 was found in 13 % (median; 0–44 %; minimum-maximum) of the perivascular cells without significant differences between 14 and 98 dpi or between SJL/J and C57BL/6 mice. PKR positive perivascular cells were only detected in one SJL/J (6 %) and one C57BL/6 mouse (25 %) at 14 dpi. In contrast, a significant higher percentage of perivascular cells (*P* = 0.036) expressed PKR in C57BL/6 mice (48 %; 38–75 %) compared to SJL/J mice (29 %; 25–36 %) at 98 dpi. However, perivascular cells were rare in C57BL/6 mice and found only in three animals.

### Immunofluorescence

**Fig. 6:**
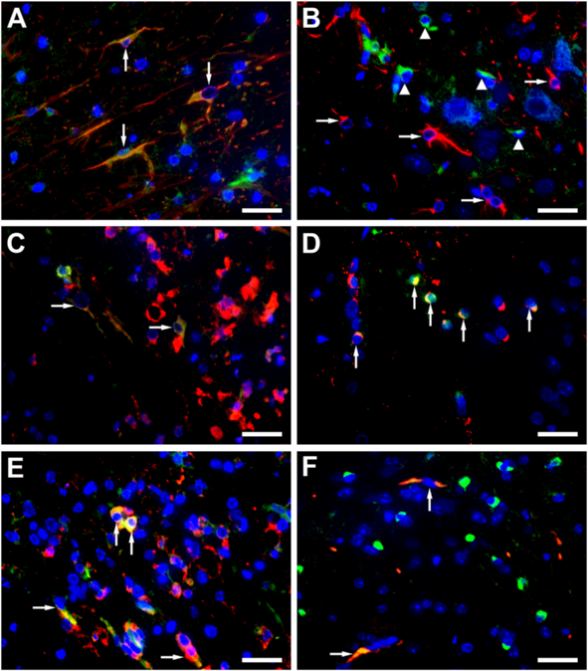
ISG15 and PKR protein expression of astrocytes, oligodendrocytes, and microglia/macrophages in spinal cord white matter lesions at 98 dpi. ISG15 (*green*) is expressed by astrocytes (*red*, **a**), oligodendrocytes (*red*, **c**), and microglia/macrophages (*red*, **e**) demonstrated by an *orange-yellow mixed color* (*arrows*). No co-localization of PKR (*green*, *arrowheads*) with GFAP (*red*, *arrows*) indicates no/low PKR expression in astrocytes (*red*, **b**), whereas co-localization of PKR (*green*) with Olig2 (*red*, **d**) and MAC-3 (*red*, **f**) demonstrates PKR expression in oligodendrocytes and microglia (*arrows*). Immunofluorescence double-staining for ISG15 and PKR with GFAP, Olig2, and MAC-3. Nuclei are stained with bisbenzimide. Bar 25 μm

Double-staining with cellular markers revealed PKR expression in Olig2-positive oligodendrocytes (Fig. 6d) and MAC-3-positive microglia/macrophages (Fig. 6f), whereas astrocytes did not express PKR (Fig. 6b). In contrast, ISG15 co-localized with GFAP confirming its astrocytic expression (Fig. 6a). In addition, few oligodendrocytes (Fig. 6c) and microglia/macrophages (Fig. 6e) expressed ISG15.

### Correlation analysis

ISG gene and protein expression was correlated to several parameters characterizing the progression of TMEV-IDD in SJL/J using Spearman’s rank correlation coefficients in order to quantify the relationship of ISGs with viral load, inflammation, and demyelination. The gene expression of ISG15, PKR, OAS1, and Mx strongly correlated with TMEV RNA, inflammatory cells including CD3^+^ T cells, CD45R^+^ B cells, and MAC3^+^ microglia/macrophages, and demyelination (*r*_*s*_ ≥ 0.6; *P* ≤ 0.05; Table 2). In addition, low but significant correlations were found between the protein expression of ISG15, PKR, and Mx and their respective gene expression, TMEV RNA, and all investigated inflammatory cells (r_s_ ≥ 0.4; *P* ≤ 0.05; Table 3). ISG15 (*r*_*s*_ = 0.3) and PKR (*r*_*s*_ = 0.4) protein expression was also significantly correlated to the extent of demyelination in the spinal cord. OAS protein expression was only significantly correlated to the overall number of inflammatory cells (*r*_*s*_ = 0.3) and the number of intralesional MAC3^+^ microglia/macrophages (*r*_*s*_ = 0.4).

**Table 2:** Spearman’s rank correlation coefficients between ISG15, PKR, OAS1, Mx1, and Mx2 gene expression with TMEV RNA, inflammation, T cells, B cells, microglia/macrophages, and demyelination in the spinal cord of SJL/J mice

ISG mRNA	TMEV RNA	Inflammation (HE)	T cells (CD3)	B cells (CD45R)	Microglia/ Macrophage (MAC3)	Demyelination (LFB)
ISG15	*0.809*	*0.832*	*0.793*	*0.714*	*0.796*	*0.660*
PKR	*0.831*	*0.797*	*0.798*	*0.709*	*0.788*	*0.638*
OAS1	*0.816*	*0.839*	*0.775*	*0.736*	*0.808*	*0.688*
Mx1	*0.708*	*0.762*	*0.645*	*0.681*	*0.775*	*0.585*
Mx2	*0.755*	*0.746*	*0.663*	*0.763*	*0.748*	*0.632*

Significant correlations are marked in italics (*P* ≤ 0.05)

**Table 3:** Spearman’s rank correlation coefficients between ISG15, PKR, OAS1, Mx1, and Mx2 protein levels with their gene expression as well as TMEV RNA, inflammation, T cells, B cells, microglia/macrophages, and demyelination in the spinal cord of SJL/J mice

ISG protein	ISG mRNA	TMEV RNA	Inflammation (HE)	T cells (CD3)	B cells (CD45R)	Microglia/ Macrophage (MAC3)	Demyelination (LFB)
ISG15	*0.499*	*0.528*	*0.484*	*0.445*	*0.363*	*0.580*	*0.316*
PKR	*0.497*	*0.417*	*0.480*	*0.444*	*0.371*	*0.434*	*0.445*
OAS1	0.208	0.118	*0.316*	0.192	0.163	*0.393*	0.212
Mx1	*0.566*	*0.566*	*0.296*	*0.604*	*0.675*	*0.659*	0.546
Mx2	0.380	*0.537*	*0.356*	*0.509*	*0.465*	*0.479*	0.505

Significant correlations are marked in italics (*P* ≤ 0.05)

## Discussion

### Impaired protein expression of interferon-stimulated genes in SJL/J mice

The present study investigated the expression of genes critically involved in the type I IFN pathway during TMEV-IDD. PRRs recognize pathogen-associated molecular patterns (PAMPs) such as viral and bacterial components and induce intrinsic cellular defenses. Double-stranded RNA formed during TMEV replication is typically identified by PKR, MDA5, and TLR3 [31, 53, 54]. PKR, MDA5, and TLR2 gene expression was moderately increased in the present study, whereas TLR3 mRNA levels were hardly affected by TMEV infection. Nevertheless, previous studies already demonstrated that TMEV induces an up-regulation of TLR2 via TLR3 signal and subsequently an activation of nuclear factor of kappa light polypeptide gene enhancer in B cells (NF-κB) causing the expression of several pro-inflammatory cytokines and chemokines including IL-1β, IL-6, CCL2 (MCP-1), and CCL5 (RANTES) [32, 33, 55, 56]. In contrast to IRF3 and IRF5, IRF7 was strongly up-regulated in the present study. Interestingly, the IRF7 protein must be continuously produced due to its short half-life [57]. In addition, IRF3 function can be blocked by the TMEV L protein [2, 15, 16]. Consequently, several PRRs might be induced by TMEV and implicated in the activation of the innate and adaptive immune response via NF-κB and IRF7. A high NF-κB expression was already described in microglia/macrophages in demyelinating TMEV lesions [55]. The activation of the type I IFN pathway by TMEV was also demonstrated by increased transcription of various ISGs including MHC class I and II genes, especially in the late phase of TMEV-IDD. Correspondingly, high correlation coefficients were found between ISG gene expression and TMEV RNA as well as inflammation and demyelination.

However, type I IFN production was blocked in the spinal cord of TMEV-infected SJL/J mice, and up-regulation of ISG transcription might depend on increased expression of ISGF3 or even IRF1, which can directly induce ISG transcription and thereby exert antiviral functions in the absence of IFN [23]. The lack of type I IFN might be related to the strong OASL1 expression found in the present study, which inhibits the translation of the potent type I IFN inducer IRF7 [58]. In addition, the increase in ISG protein expression, which only weakly correlated with its gene expression, was mainly restricted to late white matter lesions. Low ISG protein levels in the early phase of TMEV-IDD in this susceptible mouse strain indicate an intrinsic and/or virus-dependent impairment of these IFN-dependent antiviral effectors. This most likely favors virus spread within the CNS and infection of white matter glial cells. Furthermore, an impaired immune response gives the virus enough time to cause initial damage to myelin sheaths resulting in the release of myelin breakdown products. As soon as a DTH response directed against myelin sheath components has established, pro-inflammatory effects of high ISG expression within white matter lesions can even promote demyelination by enhancing antigen presentation and activating additional autoreactive immune cells. Consequently, a strong antiviral immune response in the early phase of TMEV-IDD is critical to prevent virus persistence and initiation of the demyelination process, a time point which might even define an antiviral vs. pro-inflammatory outcome of ISG actions.

### Higher ISG protein levels in C57BL/6 compared to SJL/J mice

In contrast to SJL/J mice, TMEV infection induced type I IFN transcription in C57BL/6 mice. Nevertheless, absolute IFN-β mRNA levels were higher in SJL/J compared to C57BL/6 mice at 14 dpi. RT-qPCR also revealed higher IRF7, ISG15, and PKR transcript number in TMEV-infected SJL/J at 98 dpi. However, immunohistochemistry demonstrated similar or higher protein levels of ISG15, PKR, and OAS1 in the spinal cord white and gray matter of resistant C57BL/6 mice. Moreover, ISG15 and PKR protein levels were further increased by TMEV infection. ISG15 has been reported to inhibit IRF3 degradation [59] and enhance NF-κB signaling via suppression of protein phosphatase 1B (PPM1B) [60]. Downstream targets of the transcription factors IRF3 and NF-κB including IFN-β and pro-inflammatory cytokines play an important role in the innate immune response to TMEV infection. Interestingly, the main ISG15 expressing cells were astrocytes, endothelial cells, and ependymal cells, which line the ventricular system and the central canal of the spinal cord. This expression pattern puts ISG15 in the first line of defense to restrict virus spread in the CNS via the liquorogenic route [3]. PKR is required for type I IFN production in response to TMEV by acting as cytoplasmic RNA sensor and stabilizing IFN mRNA integrity [32]. Moreover, PKR is itself up-regulated by type I IFNs and exerts direct antiviral effects [25, 61]. Similarly, OAS1 protein might act as antiviral effector in TMEV-infected C57BL/6 mice restraining virus spread in the spinal cord before demyelination starts. Nevertheless, due to the inhibition of the OAS/RNase L pathway by the TMEV L* protein [35], the antiviral activity of RNase L might be similar in TMEV-infected SJL/J and C57BL/6 mice despite different OAS1 protein levels.

## Conclusions

In conclusion, the type I IFN pathway is activated in TMEV-infected mice inducing the transcription of various ISGs. The translation of these genes seems to be impaired in susceptible SJL/J mice limiting their antiviral function. In contrast, a high constitutive expression of ISG15 and PKR proteins in the spinal cord white and gray matter, which was further increased by TMEV infection, might block the spread of TMEV in C57BL/6 mice and prevent demyelinating disease based on virus persistence.

## Additional files

Additional file 1: Table S1. Transcriptional changes of the type I and II IFN signaling pathway in TMEV- compared to mock-infected SJL/J mice. Shown are fold changes at 14, 42, 98, and 196 dpi including *P* values based on Mann-Whitney *U* tests. Bold type indicates statistically significant up-regulation (*P* < 0.05). (DOC 233 kb) Additional file 2: Figure S1. IFN-α, IFN-β, IRF7, ISG15, and PKR mRNA levels in the spinal cord of mock- and TMEV-infected SJL/J and C57BL/6 mice at 14 and 98 dpi. Shown are IFN-α (A), IFN-β (B), IRF7 (C), ISG15 (D), and PKR (E) transcript numbers using Box-and-Whisker plots and significant differences between groups based on Mann-Whitney *U* tests (**P* ≤ 0.05; ***P* ≤ 0.01). (TIF 10550 kb) Additional file 3: Figure S2. ISG15, PKR, and OAS1 protein expression in the spinal cord of mock- and TMEV-infected C57BL/6 at 14 and 98 dpi. ISG15 (A–B), PKR (C–D), and OAS1 (E–F) protein expression in spinal cord gray (A, C, E) and white matter (B, D, F). Shown is the percentage of immunopositive area using Box-and-Whisker plots and significant differences between groups based on Mann-Whitney *U* tests (**P* ≤ 0.05; ***P* ≤ 0.01). (TIF 3543 kb)

## Abbreviations

ABC: avidin-biotin-peroxidase complex
CNS: central nervous system
DAB: 3,3-diaminobenzidine
DMEM: Dulbecco’s modified Eagle medium
DTH: delayed-type hypersensitivity
eIF2α: eukaryotic initiation factor 2α
GTPase: dynamin-like large guanosine triphosphatase
IFN: interferon
IFNAR: IFN alpha and beta receptor
IL: interleukin
IRF: IFN regulatory factor
ISG: IFN-stimulated gene
ISG15: ISG of 15 kDa
ISGF: ISG factor
ISRE: IFN-stimulated response element
JAK: janus kinase
MDA5: melanoma differentiation-associated gene
MHC: major histocompatibility complex
mRNA: messenger RNA
MS: multiple sclerosis
Mx: myxovirus-resistance gene
NF-κB: nuclear factor of kappa light polypeptide gene enhancer in B cells
OAS: 2′, 5′-oligoadenylate-synthetase
ORF: open reading frame
P: phosphorylation
PFU: plaque-forming units
PPM1B: protein phosphatase 1B
PKR: protein kinase R
RNA: ribonucleic acid
RNase L: ribonuclease L
ssRNA: single-stranded RNA
STAT: signal transducer and activator of transcription
TLR: Toll-like receptor
TMEV: Theiler’s murine encephalomyelitis virus
TMEV-IDD: TMEV-induced demyelinating disease
TYK: tyrosine kinase

## References

[1] Brahic M, Bureau J-F, Michiels T (2005). The genetics of the persistent infection and demyelinating disease caused by Theiler’s virus. Annu Rev Microbiol.

[2] Stavrou S, Feng Z, Lemon SM, Roos RP (2010). Different strains of Theiler’s murine encephalomyelitis virus antagonize different sites in the type I interferon pathway. J Virol.

[3] Kummerfeld M, Seehusen F, Klein S, Ulrich R, Kreutzer R, Gerhauser I (2012). Periventricular demyelination and axonal pathology is associated with subependymal virus spread in a murine model for multiple sclerosis. Intervirology.

[4] Tsunoda I, Tanaka T, Terry EJ, Fujinami RS (2007). Contrasting roles for axonal degeneration in an autoimmune versus viral model of multiple sclerosis: when can axonal injury be beneficial?. Am J Pathol.

[5] Lipton HL, Kumar a SM, Trottier M (2005). Theiler’s virus persistence in the central nervous system of mice is associated with continuous viral replication and a difference in outcome of infection of infiltrating macrophages versus oligodendrocytes. Virus Res.

[6] Tsunoda I, Tanaka T, Saijoh Y, Fujinami RS (2007). Targeting inflammatory demyelinating lesions to sites of Wallerian degeneration. Am J Pathol.

[7] Olson JK, Miller SD (2009). The innate immune response affects the development of the autoimmune response in Theiler’s virus-induced demyelinating disease. J Immunol.

[8] Azoulay-Cayla A, Dethlefs S, Pérarnau B, Larsson-Sciard EL, Lemonnier F a, Brahic M (2000). H-2D(b-/-) mice are susceptible to persistent infection by Theiler’s virus. J Virol.

[9] Lyman M a, Myoung J, Mohindru M, Kim BS (2004). Quantitative, not qualitative, differences in CD8+ T cell responses to Theiler’s murine encephalomyelitis virus between resistant C57BL/6 and susceptible SJL/J mice. Eur J Immunol.

[10] Clatch RJ, Lipton HL, Miller SD (1987). Class II-restricted T cell responses in Theiler’s murine encephalomyelitis virus (TMEV)-induced demyelinating disease. II. Survey of host immune responses and central nervous system virus titers in inbred mouse strains. Microb Pathog.

[11] Prajeeth CK, Beineke A, Iskandar CD, Gudi V, Herder V, Gerhauser I (2014). Limited role of regulatory T cells during acute Theiler virus-induced encephalitis in resistant C57BL/6 mice. J Neuroinflammation.

[12] Kraus J, Ling AK, Hamm S, Voigt K, Oschmann P, Engelhardt B (2004). Interferon-β stabilizes barrier characteristics of brain endothelial cells in vitro. Ann Neurol.

[13] Kay M, Hojati Z, Dehghanian F (2013). The molecular study of IFNβ pleiotropic roles in MS treatment. Iran J Neurol.

[14] Ricour C, Borghese F, Sorgeloos F, Hato SV, van Kuppeveld FJM, Michiels T (2009). Random mutagenesis defines a domain of Theiler’s virus leader protein that is essential for antagonism of nucleocytoplasmic trafficking and cytokine gene expression. J Virol.

[15] Delhaye S, Van Pesch V, Michiels T (2004). The leader protein of Theiler’s virus interferes with nucleocytoplasmic trafficking of cellular proteins. J Virol..

[16] Ricour C, Delhaye S, Hato SV, Olenyik TD, Michel B, van Kuppeveld FJM (2009). Inhibition of mRNA export and dimerization of interferon regulatory factor 3 by Theiler’s virus leader protein. J Gen Virol.

[17] Moore TC, Cody L, Kumm PM, Brown DM, Petro TM (2013). IRF3 helps control acute TMEV infection through IL-6 expression but contributes to acute hippocampus damage following TMEV infection. Virus Res.

[18] Moore TC, Al-Salleeh FM, Brown DM, Petro TM (2011). IRF3 polymorphisms induce different innate anti-Theiler’s virus immune responses in RAW264.7 macrophages. Virology.

[19] Sadler AJ, Williams BRG (2008). Interferon-inducible antiviral effectors. Nat Rev Immunol.

[20] Der SD, Zhou A, Williams BR, Silverman RH (1998). Identification of genes differentially regulated by interferon alpha, beta, or gamma using oligonucleotide arrays. Proc Natl Acad Sci U S A.

[21] Jin YH, Hou W, Kim SJ, Fuller AC, Kang B, Goings G (2010). Type I interferon signals control Theiler’s virus infection site, cellular infiltration and T cell stimulation in the CNS. J Neuroimmunol.

[22] Schoggins JW (2014). Interferon-stimulated genes: roles in viral pathogenesis. Curr Opin Virol.

[23] Schoggins JW, Rice CM (2011). Interferon-stimulated genes and their antiviral effector functions. Curr Opin Virol.

[24] Harty RN, Pitha PM, Okumura A (2009). Antiviral activity of innate immune protein ISG15. J Innate Immun.

[25] Pindel A, Sadler A. The role of protein kinase R in the interferon response. 2011, 31. 10.1089/jir.2010.0099 21166592

[26] Kristiansen H, Gad HH, Eskildsen-larsen S, Despres P, Hartmann R. The oligoadenylate synthetase family : an ancient protein family with multiple antiviral activities. 2008, 31. 10.1089/jir.2010.0107 21142819

[27] Haller O, Staeheli P, Schwemmle M, Kochs G (2015). Mx GTPases: dynamin-like antiviral machines of innate immunity. Trends Microbiol.

[28] Staeheli P, Sutcliffe JG (1988). Identification of a second interferon-regulated murine Mx gene. Mol Cell Biol.

[29] Staeheli P, Grob R, Meier E, Sutcliffe JG, Haller O (1988). Influenza virus-susceptible mice carry Mx genes with a large deletion or a nonsense mutation. Mol Cell Biol.

[30] Hesse D, Sellebjerg F, Sorensen PS. Absence of MxA induction by interferon beta in patients with MS reflects complete loss of bioactivity. Neurology. 2009;372–377. 10.1212/WNL.0b013e3181b04c98 19652141

[31] Carpentier PA, Williams BR, Miller SD (2007). Distinct roles of protein kinase R and Toll-like receptor 3 in the activation of astrocytes by viral stimuli. Glia.

[32] Palma JP, Kim BS (2004). The scope and activation mechanisms of chemokine gene expression in primary astrocytes following infection with Theiler’s virus. J Neuroimmunol.

[33] Palma JP, Kwon D, Clipstone N a, Kim S, Kim BS (2003). Infection with Theiler’ s murine encephalomyelitis virus directly induces proinflammatory cytokines in primary astrocytes via NF- κB activation: potential role for the initiation of demyelinating disease infection with Theiler’s murine encephalomyeli. J Virol.

[34] Schulz O, Pichlmair A, Rehwinkel J, Rogers NC, Kato H, Takeuchi O, et al. Protein kinase R contributes to IFN-α/β production during viral infection by regulating IFN mRNA integrity. Cell Host Microbe. 2010;7:354–61. 10.1016/j.chom.2010.04.007 PMC2919169 20478537

[35] Sorgeloos F, Jha BK, Silverman RH, Michiels T. Evasion of antiviral innate immunity by Theiler’s virus L* protein through direct inhibition of RNase L. PLoS Pathog. 2013;9. 10.1371/journal.ppat.1003474 PMC3694852 23825954

[36] Kreit M, Paul S, Knoops L, De Cock A, Sorgeloos F, Michiels T (2014). Inefficient type I interferon-mediated antiviral protection of primary mouse neurons is associated with the lack of apolipoprotein l9 expression. J Virol.

[37] Markus S, Failing K, Baumgärtner W (2002). Increased expression of pro-inflammatory cytokines and lack of up-regulation of anti-inflammatory cytokines in early distemper CNS lesions. J Neuroimmunol.

[38] Navarrete-Talloni MJ, Kalkuhl A, Deschl U, Ulrich R, Kummerfeld M, Rohn K (2010). Transient peripheral immune response and central nervous system leaky compartmentalization in a viral model for multiple sclerosis. Brain Pathol.

[39] Ulrich R, Kalkuhl A, Deschl U, Baumgärtner W (2010). Machine learning approach identifies new pathways associated with demyelination in a viral model of multiple sclerosis. J Cell Mol Med.

[40] Bolstad BM, Irizarry R a, Astrand M, Speed TP (2003). A comparison of normalization methods for high density oligonucleotide array data based on variance and bias. Bioinformatics.

[41] Haist V, Ulrich R, Kalkuhl A, Deschl U, Baumgärtner W (2012). Distinct spatio-temporal extracellular matrix accumulation within demyelinated spinal cord lesions in Theiler’s murine encephalomyelitis. Brain Pathol.

[42] Hansmann F, Herder V, Kalkuhl A, Haist V, Zhang N, Schaudien D (2012). Matrix metalloproteinase-12 deficiency ameliorates the clinical course and demyelination in Theiler’s murine encephalomyelitis. Acta Neuropathol.

[43] Kreutzer M, Seehusen F, Kreutzer R, Pringproa K, Kummerfeld M, Claus P (2012). Axonopathy is associated with complex axonal transport defects in a model of multiple sclerosis. Brain Pathol.

[44] Diamond MS, Farzan M (2013). The broad-spectrum antiviral functions of IFIT and IFITM proteins. Nat Rev Immunol.

[45] Boo K-H, Yang J-S (2010). Intrinsic cellular defenses against virus infection by antiviral type I interferon. Yonsei Med J.

[46] Raddatz BB, Lehmbecker A, Kalkuhl A, Deschl U, Baumgärtner W, Ulrich R (2015). Transcriptional analysis of glial cell differentiation in postnatal murine spinal cord. Int J Dev Neurosci.

[47] Gerhauser I, Alldinger S, Ulrich R, Baumgärtner W (2005). Spatio-temporal expression of immediate early genes in the central nervous system of SJL/J mice. Int J Dev Neurosci.

[48] Vandesompele J, De Preter K, Pattyn F, Poppe B, Van Roy N, De Paepe A (2002). Accurate normalization of real-time quantitative RT-PCR data by geometric averaging of multiple internal control genes. Genome Biol.

[49] Seehusen F, Orlando E a, Wewetzer K, Baumgärtner W (2007). Vimentin-positive astrocytes in canine distemper: a target for canine distemper virus especially in chronic demyelinating lesions?. Acta Neuropathol.

[50] Ulrich R, Imbschweiler I, Kalkuhl A, Lehmbecker A, Ziege S, Kegler K (2014). Transcriptional profiling predicts overwhelming homology of schwann cells, olfactory ensheathing cells, and schwann cell-like glia. Glia.

[51] Sun Y, Lehmbecker A, Kalkuhl A, Deschl U, Sun W, Rohn K, et al. STAT3 represents a molecular switch possibly inducing astroglial instead of oligodendroglial differentiation of oligodendroglial progenitor cells in Theiler’s murine encephalomyelitis. Neuropathol Appl Neurobiol. 2014. 10.1111/nan.12133 24606160

[52] Sun Y, Lehmbecker A, Kalkuhl A, Deschl U, Sun W, Rohn K, et al. STAT3 represents a molecular switch possibly inducing astroglial instead of oligodendroglial differentiation of oligodendroglial progenitor cells in Theiler’s murine encephalomyelitis. Neuropathol Appl Neurobiol. 2015;347–370. 10.1111/nan.12133 24606160

[53] Turrin NP (2008). Central nervous system Toll-like receptor expression in response to Theiler’s murine encephalomyelitis virus-induced demyelination disease in resistant and susceptible mouse strains. Virol J.

[54] Jin Y-H, Kim SJ, So EY, Meng L, Colonna M, Kim BS (2012). Melanoma differentiation-associated gene 5 is critical for protection against Theiler’s virus-induced demyelinating disease. J Virol.

[55] Gerhauser I, Ulrich R, Alldinger S, Baumgärtner W (2007). Induction of activator protein-1 and nuclear factor-kappaB as a prerequisite for disease development in susceptible SJL/J mice after theiler murine encephalomyelitis. J Neuropathol Exp Neurol.

[56] So EY, Kim BS (2009). Theiler’s virus infection induces TLR3-dependent upregulation of TLR2 critical for proinflammatory cytokine production. Glia.

[57] Taniguchi T, Takaoka A (2001). A weak signal for strong responses: interferon-alpha/beta revisited. Nat Rev Mol Cell Biol.

[58] Lee MS, Kim B, Oh GT, Kim Y-J (2013). OASL1 inhibits translation of the type I interferon-regulating transcription factor IRF7. Nat Immunol.

[59] Lu G, Reinert JT, Pitha-Rowe I, Okumura a, Kellum M, Knobeloch KP (2006). ISG15 enhances the innate antiviral response by inhibition of IRF-3 degradation. Cell Mol Biol.

[60] Takeuchi T, Kobayashi T, Tamura S, Yokosawa H (2006). Negative regulation of protein phosphatase 2Cbeta by ISG15 conjugation. FEBS Lett.

[61] Ank N, West H, Bartholdy C, Eriksson K, Thomsen AR, Paludan SR (2006). Lambda interferon (IFN-lambda), a type III IFN, is induced by viruses and IFNs and displays potent antiviral activity against select virus infections in vivo. J Virol.

