# Supplementary material for: Interferon-stimulated genes—essential antiviral effectors implicated in resistance to Theiler’s virus-induced demyelinating disease
Source: J Neuroinflammation. 2015 Dec 24;12:242. doi: 10.1186/s12974-015-0462-x (PMC4690264; doi:10.1186/s12974-015-0462-x)
Supplement: Additional file 1: Table S1. — Transcriptional changes of the type I and II IFN signaling pathway in TMEV- compared to mock-infected SJL/J mice. Shown are fold changes at 14, 42, 98, and 196 dpi including P values based on Mann-Whitney U tests. Bold type indicates statistically significant up-regulation (P < 0.05). (DOC 233 kb) [file 12974_2015_462_MOESM1_ESM.doc]

**Additional file 1**

**Table S1 Transcriptional changes of the type I and II IFN signaling pathway in TMEV- compared to mock-infected SJL/J mice.** Shown are fold changes at 14, 42, 98, and 196 dpi including p values based on Mann-Whitney-U tests. Bold type indicates statistically significant up-regulation (*P* < 0.05).

| **Probe Set ID** | **Gene Symbol** | **Gene Title** | **14 dpi** | | **42 dpi** | | **98 dpi** | | **196 dpi** | |
| --- | --- | --- | --- | --- | --- | --- | --- | --- | --- | --- |
|  |  |  | **Fold change** | ***P*-value** | **Fold change** | ***P*-value** | **Fold change** | ***P*-value** | **Fold change** | ***P*-value** |
| **Pattern Recognition Receptors** | | | | | | | | | | |
| 1456890_at | Ddx58 (RIG-I) | DEAD (Asp-Glu-Ala-Asp) box polypeptide 58 | **1.63** | 0.026 | **3.17** | 0.002 | **3.53** | 0.004 | **2.24** | 0.002 |
| 1440866_at | Eif2ak2 (PKR) | Eukaryotic translation initiation factor 2-alpha kinase 4 | **1.69** | 0.015 | **3.04** | 0.002 | **3.73** | 0.004 | **2.18** | 0.002 |
| 1426276_at | Ifih1 (MDA5) | Interferon induced with helicase C domain 1 | **2.20** | 0.002 | **4.86** | 0.002 | **5.77** | 0.004 | **3.51** | 0.002 |
| 1449049_at | Tlr1 | Toll-like receptor 1 | 1.25 | 0.132 | **2.29** | 0.002 | **2.81** | 0.004 | **2.51** | 0.002 |
| 1419132_at | Tlr2 | Toll-like receptor 2 | **1.77** | 0.004 | **2.66** | 0.002 | **3.31** | 0.004 | **2.94** | 0.002 |
| 1422781_at | Tlr3 | Toll-like receptor 3 | 1.18 | 0.180 | **1.37** | 0.041 | **1.89** | 0.004 | **1.38** | 0.002 |
| 1418162_at | Tlr4 | Toll-like receptor 4 | 1.00 | 0.898 | 1.20 | 0.180 | **1.41** | 0.030 | **2.01** | 0.002 |
| 1422010_at | Tlr7 | Toll-like receptor 7 | 1.18 | 0.238 | **1.87** | 0.002 | **2.47** | 0.004 | **2.43** | 0.002 |
| 1450267_at | Tlr8 | Toll-like receptor 8 | -1.03 | 0.571 | 1.03 | 1.000 | 1.01 | 0.628 | 1.09 | 0.305 |
| 1457753_at | Tlr13 | Toll-like receptor 13 | **1.33** | 0.041 | **2.50** | 0.002 | **3.46** | 0.004 | **3.27** | 0.002 |
| **Interferon Regulatory Factors** | | | | | | | | | | |
| 1448436_a_at | Irf1 | Interferon regulatory factor 1 | **1.51** | 0.009 | **3.47** | 0.002 | **4.40** | 0.004 | **3.11** | 0.002 |
| 1418265_s_at | Irf2 | Interferon regulatory factor 2 | 1.08 | 0.180 | **1.26** | 0.004 | 1.35 | 0.052 | **1.26** | 0.026 |
| 1426111_x_at | Irf3 | Interferon regulatory factor 3 | 1.07 | 0.180 | 1.06 | 0.240 | 1.09 | 0.126 | **1.18** | 0.002 |
| 1421173_at | Irf4 | Interferon regulatory factor 4 | 1.09 | 0.310 | **2.21** | 0.002 | **3.52** | 0.004 | **4.70** | 0.002 |
| 1460231_at | Irf5 | Interferon regulatory factor 5 | 1.07 | 0.394 | **1.40** | 0.002 | **1.81** | 0.004 | **1.57** | 0.002 |
| 1418301_at | Irf6 | Interferon regulatory factor 6 | -1.21 | 0.310 | 1.07 | 0.589 | -1.12 | 0.329 | 1.29 | 0.132 |
| 1417244_a_at | Irf7 | Interferon regulatory factor 7 | **2.66** | 0.004 | **10.12** | 0.002 | **11.42** | 0.004 | **4.46** | 0.002 |
| 1416714_at | Irf8 | Interferon regulatory factor 8 | **2.07** | 0.002 | **3.80** | 0.002 | **5.87** | 0.004 | **3.77** | 0.002 |
| 1427705_a_at | Nfkb1 | Nuclear factor of kappa light polypeptide gene enhancer in B cells 1, p105 | **1.14** | 0.041 | **1.14** | 0.004 | **1.20** | 0.009 | **1.10** | 0.009 |
| 1419536_a_at | Rela | v-rel reticuloendotheliosis viral oncogene homolog A (avian) | **1.11** | 0.026 | **1.11** | 0.041 | 1.18 | 0.052 | **1.15** | 0.041 |
| **Type I/II Interferons** | | | | | | | | | | |
| 1450564_x_at | Ifna1 | Interferon alpha 1 | 1.04 | 0.485 | -1.10 | 0.394 | -1.06 | 0.537 | -1.08 | 0.180 |
| 1423028_at | Ifna2 | Interferon alpha 2 | -1.05 | 0.394 | 1.00 | 0.937 | 1.00 | 0.931 | -1.05 | 0.240 |
| 1422408_at | Ifna4 | Interferon alpha 4 | 1.02 | 0.818 | -1.04 | 0.937 | -1.08 | 0.329 | -1.02 | 0.485 |
| 1450614_x_at | Ifna5 | Interferon alpha 5 | -1.08 | 0.485 | -1.15 | 0.132 | -1.09 | 0.429 | -1.00 | 1.000 |
| 1422403_at | Ifna7 | Interferon alpha 7 | 1.01 | 0.937 | 1.05 | 0.699 | 1.05 | 0.329 | 1.03 | 0.818 |
| 1422406_at | Ifna9 | Interferon alpha 9 | -1.05 | 0.485 | -1.11 | 0.240 | -1.03 | 0.662 | 1.00 | 0.818 |
| 1422332_at | Ifna11 | Interferon alpha 11 | -1.01 | 0.937 | -1.12 | 0.394 | 1.03 | 0.537 | 1.01 | 0.937 |
| 1422305_at | Ifnb1 | Interferon beta 1, fibroblast | 1.02 | 1.000 | 1.07 | 0.589 | -1.08 | 0.792 | -1.11 | 0.132 |
| 1425947_at | Ifng | Interferon gamma | 1.16 | 0.240 | **2.02** | 0.002 | **2.69** | 0.004 | 1.59 | 0.065 |
| **Type I/II Interferon Receptors** | | | | | | | | | | |
| 1449026_at | Ifnar1 | Interferon (alpha and beta) receptor 1 | 1.01 | 0.818 | -1.02 | 0.485 | **1.14** | 0.030 | **1.15** | 0.009 |
| 1440169_x_at | Ifnar2 | Interferon (alpha and beta) receptor 2 | 1.10 | 0.394 | **1.51** | 0.002 | **1.72** | 0.004 | **1.50** | 0.002 |
| 1448167_at | Ifngr1 | Interferon gamma receptor 1 | 1.03 | 0.093 | 1.12 | 0.065 | **1.53** | 0.004 | **1.38** | 0.002 |
| 1423558_at | Ifngr2 | Interferon gamma receptor 2 | -1.12 | 0.093 | -1.02 | 0.937 | 1.03 | 0.931 | -1.02 | 0.589 |
| **Signal Transducers** | | | | | | | | | | |
| 1421322_a_at | Irf9 | Interferon regulatory factor 9 | **2.53** | 0.002 | **4.92** | 0.002 | **4.72** | 0.004 | **3.21** | 0.002 |
| 1433805_at | Jak1 | Janus kinase 1 | 1.01 | 0.589 | 1.02 | 0.394 | -1.04 | 0.537 | 1.06 | 0.041 |
| 1421066_at | Jak2 | Janus kinase 2 | 1.11 | 0.132 | **1.19** | 0.004 | **1.31** | 0.004 | **1.21** | 0.009 |
| 1450446_a_at | Socs1 | Suppressor of cytokine signaling 1 | 1.01 | 0.818 | **1.31** | 0.015 | 1.48 | 0.082 | 1.14 | 0.132 |
| 1418507_s_at | Socs2 | Suppressor of cytokine signaling 2 | -1.13 | 0.240 | **1.18** | 0.041 | 1.27 | 0.082 | **1.38** | 0.026 |
| 1450033_a_at | Stat1 | Signal transducer and activator of transcription 1 | **3.26** | 0.002 | **10.58** | 0.002 | **15.39** | 0.004 | **9.57** | 0.002 |
| 1421911_at | Stat2 | Signal transducer and activator of transcription 2 | **1.39** | 0.002 | **2.49** | 0.002 | **3.30** | 0.004 | **2.03** | 0.002 |
| 1417306_at | Tyk2 | Tyrosine kinase 2 | 1.15 | 0.093 | **1.12** | 0.026 | **1.32** | 0.004 | **1.19** | 0.002 |
| **Interferon-Dependent Antiviral Effectors** | | | | | | | | | | |
| 1439276_at | Adar | Adenosine deaminase, RNA-specific | **2.28** | 0.015 | **1.57** | 0.041 | **1.49** | 0.030 | 1.27 | 0.305 |
| 1417470_at | Apobec3 | Apolipoprotein B mRNA editing enzyme, catalytic polypeptide 3 | -1.09 | 0.065 | **1.23** | 0.026 | **1.27** | 0.009 | **1.41** | 0.002 |
| 1424921_at | Bst2 (Tetherin) | Bone marrow stromal cell antigen 2 | **3.47** | 0.002 | **12.71** | 0.002 | **15.60** | 0.004 | **7.70** | 0.002 |
| 1440299_at | Mb21d1 (C6orf150) | Mab-21 domain containing 1 | 1.13 | 0.180 | **1.26** | 0.004 | **1.42** | 0.004 | 1.08 | 0.180 |
| 1418283_at | Cldn4 | Claudin 4 | -1.06 | 0.305 | 1.02 | > 0.999 | -1.07 | 0.749 | 1.03 | 0.368 |
| 1449227_at | Ch25h | Cholesterol 25-hydroxylase | **1.26** | 0.015 | **2.25** | 0.002 | **2.82** | 0.004 | **2.76** | 0.002 |
| 1428306_at | Ddit4 | DNA-damage-inducible transcript 4 | -1.24 | 0.305 | -1.12 | 0.571 | -1.01 | 0.900 | -1.01 | 0.898 |
| 1437773_x_at | Ddx17 | DEAD (Asp-Glu-Ala-Asp) box polypeptide 17 | 1.06 | 0.1780 | -1.02 | 0.788 | 1.06 | 0.424 | -1.00 | 0.898 |
| 1451777_at | Ddx60 | DEAD (Asp-Glu-Ala-Asp) box polypeptide 60 | **2.02** | 0.009 | **5.27** | 0.002 | **5.69** | 0.004 | **3.77** | 0.002 |
| 1435906_x_at | Gbp2 | Guanylate binding protein 2 | **2.66** | 0.009 | **10.19** | 0.002 | **11.15** | 0.004 | **7.33** | 0.002 |
| 1433930_at | Hpse | Heparanase | 1.08 | 0.387 | **2.80** | 0.002 | **8.53** | 0.004 | **10.22** | 0.002 |
| 1422476_at | Ifi30 | interferon gamma inducible protein 30 | **1.37** | 0.041 | **3.14** | 0.002 | **4.61** | 0.004 | **3.02** | 0.002 |
| 1445897_s_at | Ifi35 | Interferon-induced protein 35 | **2.43** | 0.009 | **6.06** | 0.002 | **9.67** | 0.004 | **5.81** | 0.002 |
| 1425917_at | Ifi44l | Interferon-induced protein 44 like | **1.46** | 0.041 | 2.18 | 0.675 | 1.66 | > 0.999 | 1.98 | 0.238 |
| 1417292_at | Ifi47 | Interferon gamma inducible protein 47 | **5.13** | 0.002 | **21.28** | 0.002 | **31.22** | 0.004 | **20.16** | 0.002 |
| 1421551_s_at | Ifi202b | Interferon activated gene 202B | **3.80** | 0.002 | **14.73** | 0.002 | **18.28** | 0.004 | **11.13** | 0.002 |
| 1442828_at | Ifi203 | Interferon activated gene 203 | -1.05 | 0.818 | 1.09 | 0.093 | 1.10 | 0.126 | -1.00 | 0.937 |
| 1450783_at | Ifit1 (Isg56) | Interferon-induced protein with tetratricopeptide repeats 1 | **6.50** | 0.002 | **18.68** | 0.002 | **19.51** | 0.004 | **8.96** | 0.002 |
| 1418293_at | Ifit2 (Isg54) | Interferon-induced protein with tetratricopeptide repeats 2 | **2.01** | 0.004 | **4.58** | 0.002 | **5.34** | 0.004 | **2.68** | 0.002 |
| 1449025_at | Ifit3 (Isg60) | Interferon-induced protein with tetratricopeptide repeats 3 | **3.65** | 0.002 | **10.87** | 0.002 | **11.96** | 0.004 | **5.23** | 0.002 |
| 1424254_at | Ifitm1 | Interferon induced transmembrane protein 1 | 1.31 | 0.305 | **1.74** | 0.009 | **1.57** | 0.017 | **1.82** | 0.004 |
| 1417460_at | Ifitm2 | Interferon induced transmembrane protein 2 | 1.33 | 0.093 | **1.71** | 0.002 | **1.53** | 0.009 | **1.85** | 0.002 |
| 1423754_at | Ifitm3 | Interferon induced transmembrane protein 3 | **2.15** | 0.004 | **4.17** | 0.002 | **4.61** | 0.004 | **3.48** | 0.002 |
| 1440216_at | Ifitm5 | Interferon induced transmembrane protein 5 | -1.04 | 0.788 | -1.12 | 0.305 | -1.04 | 0.515 | 1.04 | 0.788 |
| 1440865_at | Ifitm6 | Interferon induced transmembrane protein 6 | 1.01 | 0.898 | **1.40** | 0.009 | **1.63** | 0.009 | **1.49** | 0.009 |
| 1453917_at | Ifitm7 | Interferon induced transmembrane protein 7 | 1.10 | 0.238 | 1.09 | 0.305 | -1.00 | 0.749 | -1.16 | 0.065 |
| 1455239_at | Ifitm10 | Interferon induced transmembrane protein 10 | -1.10 | 0.387 | **-1.13** | 0.041 | -1.15 | 0.052 | **-1.15** | 0.041 |
| 1431591_s_at | Isg15 | ISG15 ubiquitin-like modifier | **3.23** | 0.009 | **11.58** | 0.002 | **15.71** | 0.004 | **6.59** | 0.002 |
| 1419569_a_at | Isg20 | Interferon-stimulated protein | **1.52** | 0.015 | **3.76** | 0.002 | **5.97** | 0.004 | **3.42** | 0.002 |
| 1434364_at | Map3k14 (Nik) | Mitogen-activated protein kinase kinase kinase 14 | -1.07 | 0.238 | -1.02 | 0.898 | **1.25** | 0.030 | **1.35** | 0.026 |
| 1416380_at | Mov10 | Moloney leukemia virus 10 | 1.17 | 0.132 | **1.29** | 0.007 | **1.58** | 0.004 | **1.40** | 0.002 |
| 1451905_a_at | Mx1 | Myxovirus (influenza virus) resistance 1 | 1.34 | 0.093 | **2.11** | 0.002 | **3.85** | 0.004 | **1.88** | 0.002 |
| 1419676_at | Mx2 | Myxovirus (influenza virus) resistance 2 | 1.24 | 0.132 | **1.61** | 0.002 | **1.76** | 0.004 | **1.31** | 0.009 |
| 1417190_at | Nampt (Pbef1) | Nicotinamide phosphoribosyltransferase | 1.11 | 0.238 | **1.50** | 0.002 | **1.87** | 0.004 | **1.59** | 0.002 |
| 1451050_at | Nt5c3 | 5'-nucleotidase, cytosolic III | -1.01 | 0.675 | -1.08 | 0.132 | 1.07 | 0.178 | -1.05 | 0.180 |
| 1424775_at | Oas1a | 2'-5' oligoadenylate synthetase 1A | **2.71** | 0.004 | **7.92** | 0.002 | **11.07** | 0.004 | **6.73** | 0.002 |
| 1425119_at | Oas1b | 2'-5' oligoadenylate synthetase 1B | **1.29** | 0.002 | **1.65** | 0.002 | **1.70** | 0.004 | **1.33** | 0.026 |
| 1418686_at | Oas1c | 2'-5' oligoadenylate synthetase 1C | -1.11 | 0.180 | 1.16 | 0.093 | 1.22 | 0.177 | 1.11 | 0.394 |
| 1416847_s_at | Oas1d /// Oas1e | 2'-5' oligoadenylate synthetase 1D /// 2'-5' oligoadenylate synthetase 1E | -1.09 | 0.065 | -1.03 | 0.937 | -1.07 | 0.537 | -1.12 | 0.065 |
| 1424536_at | Oas1e | 2'-5' oligoadenylate synthetase 1E | 1.02 | 0.818 | -1.06 | 0.132 | -1.07 | 0.329 | -1.04 | 0.589 |
| 1442389_at | Oas1f | 2'-5' oligoadenylate synthetase 1F | -1.02 | 0.589 | **-1.17** | 0.009 | 1.03 | 0.792 | 1.02 | 0.818 |
| 1425049_at | Oas1h | 2'-5' oligoadenylate synthetase 1H | 1.09 | 0.818 | 1.06 | 0.937 | -1.03 | 0.662 | 1.13 | 0.180 |
| 1425065_at | Oas2 | 2'-5' oligoadenylate synthetase 2 | **1.32** | 0.009 | **2.26** | 0.002 | **2.59** | 0.004 | **2.08** | 0.002 |
| 1425374_at | Oas3 | 2'-5' oligoadenylate synthetase 3 | 1.01 | 0.937 | **1.59** | 0.004 | **1.75** | 0.004 | 1.23 | 0.180 |
| 1424339_at | Oasl1 | 2'-5' oligoadenylate synthetase-like 1 | **1.61** | 0.004 | **2.81** | 0.002 | **3.65** | 0.004 | **1.99** | 0.002 |
| 1453196_a_at | Oasl2 | 2'-5' oligoadenylate synthetase-like 2 | **6.70** | 0.002 | **25.78** | 0.002 | **29.16** | 0.004 | **12.93** | 0.002 |
| 1425214_at | P2ry6 | Pyrimidinergic receptor P2Y, G-protein coupled, 6 | **1.81** | 0.004 | **2.88** | 0.002 | **4.12** | 0.004 | **3.41** | 0.002 |
| 1456103_at | Pml (Trim19) | Promyelocytic leukemia | **1.27** | 0.026 | 1.60 | 0.009 | **1.95** | 0.004 | **1.55** | 0.002 |
| 1426604_at | Rnasel | Ribonuclease L (2', 5'-oligoisoadenylate synthetase-dependent) | -1.16 | 0.132 | 1.37 | 0.065 | **1.54** | 0.004 | **1.29** | 0.026 |
| 1421009_at | Rsad2 (Viperin) | Radical S-adenosyl methionine domain containing 2 | **2.33** | 0.004 | **6.02** | 0.002 | **8.64** | 0.004 | **3.90** | 0.002 |
| 1418580_at | Rtp4 | Receptor transporter protein 4 | **4.40** | 0.002 | **11.88** | 0.002 | **12.65** | 0.004 | **7.25** | 0.002 |
| 1418131_at | Samhd1 | SAM domain and HD domain, 1 | **1.76** | 0.004 | **3.43** | 0.002 | **3.83** | 0.004 | **2.41** | 0.002 |
| 1420697_at | Slc15a3 | Solute carrier family 15, member 3 | **1.52** | 0.015 | **4.60** | 0.002 | **5.95** | 0.004 | **4.56** | 0.002 |
| 1424776_a_at | Slc25a28 | Solute carrier family 25, member 28 | 1.05 | 0.474 | 1.08 | 0.065 | -1.04 | 0.416 | -1.05 | 0.093 |
| 1427917_s_at | Ssbp3 | Single-stranded DNA binding protein 3 | 1.01 | 0.675 | -1.03 | 0.180 | **-1.15** | 0.004 | **-1.07** | 0.026 |
| 1450672_a_at | Trex1 (Atrip) | Three prime repair exonuclease 1 (ATR interacting protein) | 1.20 | 0.093 | **1.87** | 0.002 | **2.26** | 0.004 | **1.59** | 0.002 |
| 1442693_at | Trim5 | Tripartite motif-containing 5 | 1.33 | 0.093 | **1.85** | 0.002 | **2.44** | 0.004 | **2.63** | 0.002 |
| 1425974_a_at | Trim25 | Tripartite motif-containing 25 | **2.18** | 0.004 | **3.49** | 0.002 | **4.03** | 0.004 | **3.32** | 0.002 |
| 1433832_at | Sun2 (Unc84b) | Sad1 and UNC84 domain containing 2 | 1.02 | 0.571 | **1.06** | 0.041 | 1.08 | 0.126 | **1.09** | 0.004 |
| 1436183_at | Zc3hav1 (Zap) | Zinc finger CCCH type, antiviral 1 | **2.13** | 0.002 | **3.61** | 0.002 | **3.69** | 0.004 | **2.99** | 0.002 |
| **Major Histocompatibility (MHC) Genes Class I/II** | | | | | | | | | | |
| 1425519_a_at | Cd74 | CD74 antigen (invariant polypeptide of major histocompatibility complex, class II antigen-associated) | **5.31** | 0.002 | **19.76** | 0.002 | **21.74** | 0.004 | **18.91** | 0.002 |
| 1451784_x_at | H2-D1 (MHC-Ia) | Histocompatibility 2, D region locus 1 | **5.58** | 0.002 | **8.03** | 0.002 | **9.78** | 0.004 | **9.42** | 0.002 |
| 1451931_x_at | H2-K1 (MHC-Ia) | Histocompatibility 2, K1, K region | **5.42** | 0.002 | **7.45** | 0.002 | **9.70** | 0.004 | **8.56** | 0.002 |
| 1421358_at | H2-M3 (MHC-Ib) | Histocompatibility 2, M region locus 3 | **1.62** | 0.002 | **2.80** | 0.002 | **3.48** | 0.004 | **3.25** | 0.002 |
| 1430802_at | H2-Q5 (MHC-Ib) | histocompatibility 2, Q region locus 5 | **2.46** | 0.002 | **9.75** | 0.002 | **13.88** | 0.004 | **10.7** | 0.002 |
| 1422160_at | H2-T24 (MHC-Ib) | histocompatibility 2, T region locus 24 | 1.17 | 0.238 | **2.05** | 0.009 | **2.40** | 0.004 | **2.00** | 0.009 |
| 1452431_s_at | H2-Aa (MHC-IIa) | histocompatibility 2, class II antigen A, alpha | **5.49** | 0.002 | **29.30** | 0.002 | **37.55** | 0.004 | **29.10** | 0.002 |
| 1417025_at | H2-Eb1 (MHC-IIa) | histocompatibility 2, class II antigen E beta | **5.11** | 0.002 | **20.11** | 0.002 | **28.18** | 0.004 | **21.00** | 0.002 |
| 1422527_at | H2-DMa (MHC-IIb) | histocompatibility 2, class II, locus DMa | **1.31** | 0.026 | **2.93** | 0.002 | **4.23** | 0.004 | **3.22** | 0.002 |
| 1419297_at | H2-Oa (MHC-IIb) | histocompatibility 2, O region alpha locus | 1.25 | 0.065 | **2.78** | 0.002 | **4.39** | 0.004 | **2.79** | 0.002 |
