# Supplementary figures and images for: Interferon-stimulated genes—essential antiviral effectors implicated in resistance to Theiler’s virus-induced demyelinating disease
Source: J Neuroinflammation. 2015 Dec 24;12:242. doi: 10.1186/s12974-015-0462-x (PMC4690264; doi:10.1186/s12974-015-0462-x)

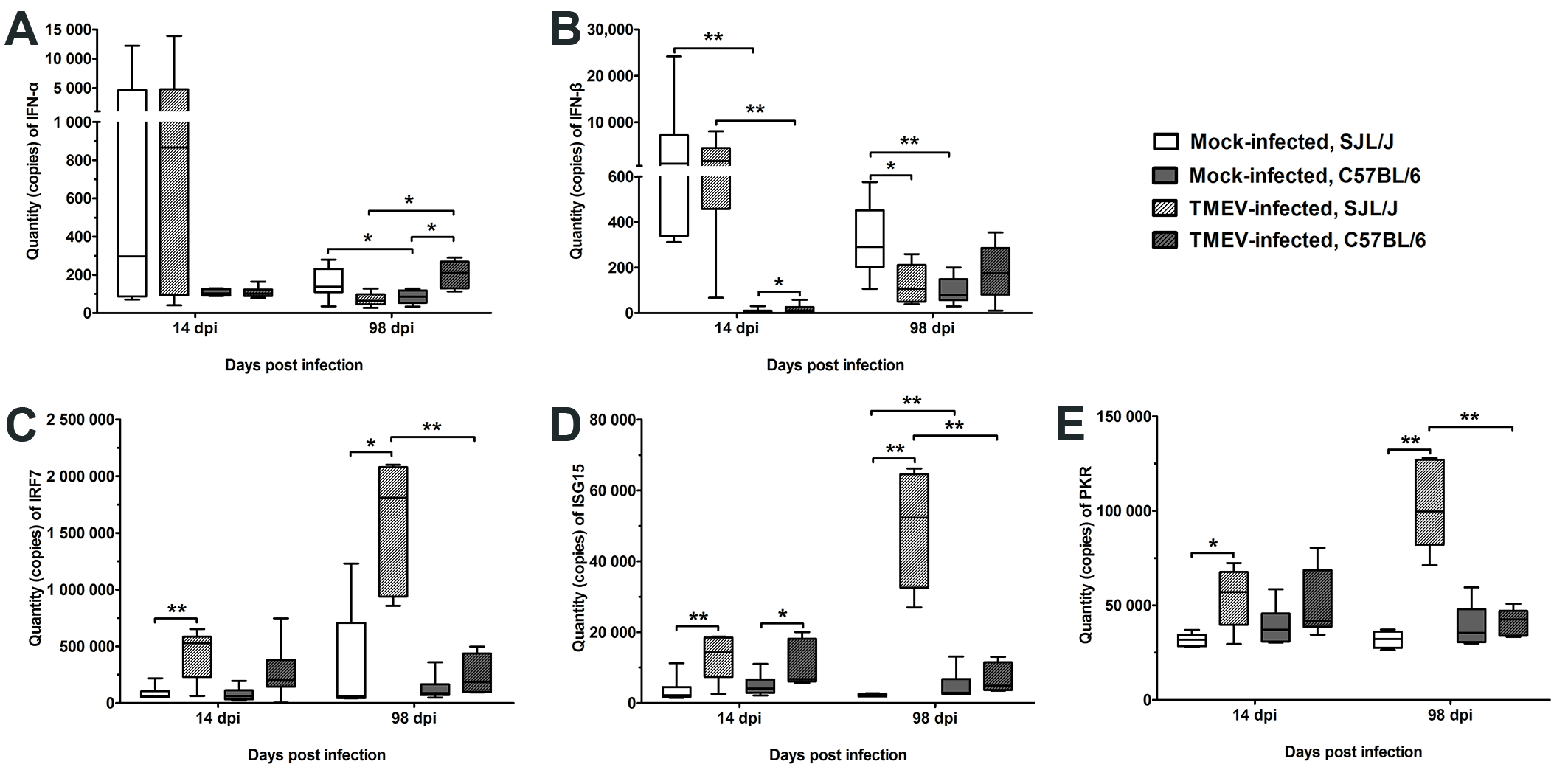

Supplement: Additional file 2: Figure S1. — IFN-α, IFN-β, IRF7, ISG15, and PKR mRNA levels in the spinal cord of mock- and TMEV-infected SJL/J and C57BL/6 mice at 14 and 98 dpi. Shown are IFN-α (A), IFN-β (B), IRF7 (C), ISG15 (D), and PKR (E) transcript numbers using Box-and-Whisker plots and significant differences between groups based on Mann-Whitney U tests (*P ≤ 0.05; **P ≤ 0.01). (TIF 10550 kb) [file 12974_2015_462_MOESM2_ESM.tif]

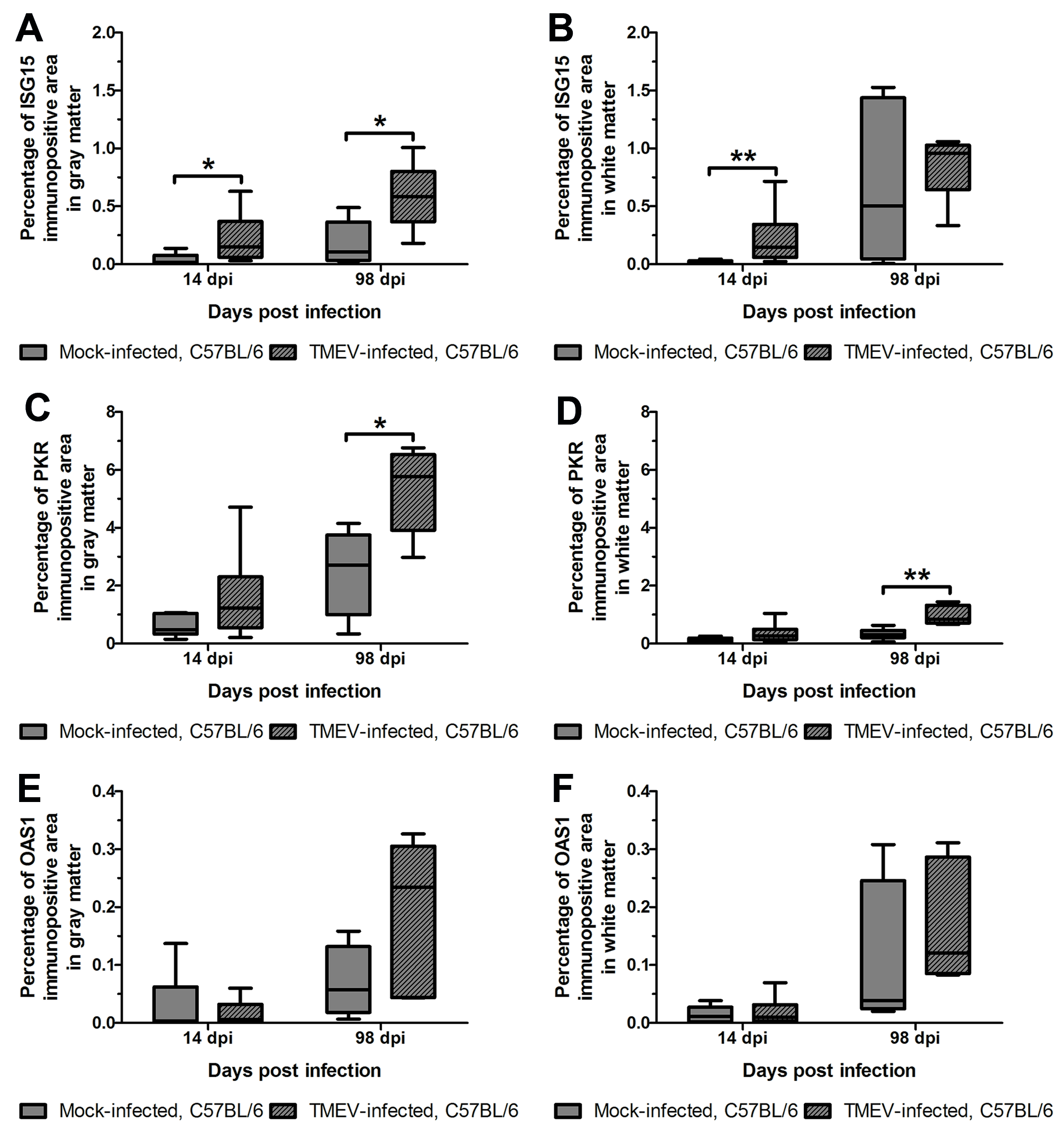

Supplement: Additional file 3: Figure S2. — ISG15, PKR, and OAS1 protein expression in the spinal cord of mock- and TMEV-infected C57BL/6 at 14 and 98 dpi. ISG15 (A–B), PKR (C–D), and OAS1 (E–F) protein expression in spinal cord gray (A, C, E) and white matter (B, D, F). Shown is the percentage of immunopositive area using Box-and-Whisker plots and significant differences between groups based on Mann-Whitney U tests (*P ≤ 0.05; **P ≤ 0.01). (TIF 3543 kb) [file 12974_2015_462_MOESM3_ESM.tif]
